# A gene expression signature identifying transient DNMT1 depletion as a causal factor of cancer-germline gene activation in melanoma

**DOI:** 10.1186/s13148-015-0147-4

**Published:** 2015-10-26

**Authors:** Julie Cannuyer, Aurélie Van Tongelen, Axelle Loriot, Charles De Smet

**Affiliations:** Group of Genetics and Epigenetics, de Duve Institute, Université Catholique de Louvain, Brussels, Belgium

## Abstract

**Background:**

Many human tumors show aberrant activation of a group of germline-specific genes, termed cancer-germline (CG) genes, several of which appear to exert oncogenic functions. Although activation of CG genes in tumors has been linked to promoter DNA demethylation, the mechanisms underlying this epigenetic alteration remain unclear. Two main processes have been proposed: awaking of a gametogenic program directing demethylation of target DNA sequences via specific regulators, or general deficiency of DNA methylation activities resulting from mis-targeting or down-regulation of the DNMT1 methyltransferase.

**Results:**

By the analysis of transcriptomic data, we searched to identify gene expression changes associated with CG gene activation in melanoma cells. We found no evidence linking CG gene activation with differential expression of gametogenic regulators. Instead, CG gene activation correlated with decreased expression of a set of mitosis/division-related genes (ICCG genes). Interestingly, a similar gene expression signature was previously associated with depletion of DNMT1. Consistently, analysis of a large set of melanoma tissues revealed that DNMT1 expression levels were often lower in samples showing activation of multiple CG genes. Moreover, by using immortalized melanocytes and fibroblasts carrying an inducible anti-DNMT1 small hairpin RNA (shRNA), we demonstrate that transient depletion of DNMT1 can lead to long-term activation of CG genes and repression of ICCG genes at the same time. For one of the ICCG genes (*CDCA7L*), we found that its down-regulation in melanoma cells was associated with deposition of repressive chromatin marks, including H3K27me3.

**Conclusions:**

Together, our observations point towards transient DNMT1 depletion as a causal factor of CG gene activation in vivo in melanoma.

**Electronic supplementary material:**

The online version of this article (doi:10.1186/s13148-015-0147-4) contains supplementary material, which is available to authorized users.

## Background

Cancer development is a multistep process during which neoplastic cells accumulate new properties that progressively increase their malignant behavior [1]. This stepwise progression is in part driven by the acquisition of genetic mutations that modify the function of cancer-related proteins [2]. Other driving forces are epigenetic alterations, which affect both DNA and histone modifications and lead to reshaping of chromatin structures, thereby permitting gene expression adaptations that favor cancer progression [3].

One common epigenetic alteration in human tumors concerns DNA methylation, a chemical modification that affects cytosines in CpG sequences and is associated with transcriptional repression [4]. Lineage-specific DNA methylation patterns, which are established during embryonic development, are in general faithfully maintained in differentiated adult cells. Enzymes involved in these processes are the de novo methyltransferases DNMT3A and DNMT3B, as well as the DNA methylation maintenance methyltransferase DNMT1 [5]. In most tumor cells, genome methylation patterns become profoundly altered. Both gains (hypermethylation) and losses (hypomethylation) of DNA methylation are observed within the same tumor cell. In many cases, hypermethylation affects the promoter of tumor-suppressor genes and leads therefore to loss of tumor-suppressive functions [3]. Compared with DNA hypermethylation, DNA hypomethylation is more widespread in cancer genomes, as it affects repeated sequences which are dispersed throughout all chromosomes [6]. DNA hypomethylation was shown to promote tumor development at least in part by increasing genome instability [7].

Another frequent target of DNA hypomethylation in tumors comprises a particular group of germline-specific genes, including about 50 genes or gene families in human, which were grouped under the term “cancer-germline” (CG) genes [8]. A common characteristic of CG genes is that their repression in somatic tissues primarily relies on DNA methylation, which appears to act as a dominant component of transcriptional regulation for these genes [9, 10]. As a result, while CG genes are completely silenced in normal somatic tissues, they become activated, often concomitantly, in tumors with extensive genome hypomethylation. Aberrant activation of CG genes is observed in a wide variety of tumors, including lung, head and neck, œsophagal, bladder, and prostate carcinoma, as well as melanoma [11]. CG genes were initially identified because their activation in tumor cells generates the expression of tumor-specific antigens, which can be recognized by cytolytic T lymphocytes [12]. Therapeutical anti-cancer vaccinations directed against such antigens are currently being tested in the clinic. More recently, several CG genes were also found to display oncogenic properties, thereby suggesting that several of these genes may contribute to tumor progression [13, 14].

The process leading to DNA demethylation and subsequent activation of CG genes in tumors is still unclear. Two main possibilities have been envisaged. One involves induction of a gametogenic program in cancer cells, which would result from aberrant reactivation of critical master regulators of germ cell development [11, 15]. In association with TET methylcytosine oxidizing enzymes, such regulators could elicit demethylation of target DNA sequences [16–18]. The other possible cause of CG gene hypomethylation in tumors relies on a general defect in DNA methylation maintenance activities, and several mechanisms that might cause such type of epigenetic imbalance have been proposed. For instance, mis-localization of DNMT1 in cancer cells, resulting from either impaired recruitment to replication forks, disrupted interaction with partner proteins, or translocation into a stress-induced protein complex, has been reported [19–21]. Excessive proliferation and deficiency in the production of the methyl donor S-adenosylmethionine were also suggested as a possible cause of DNA demethylation in cancer [22]. Other studies revealed down-regulation of DNMT1 expression in tumor cells, via increased abundance of a regulatory miRNA (miR29b) or over-expression of a partner protein (UHRF1) that induces DNMT1 destabilization [23, 24]. It is expected, however, that the process leading to DNA demethylation operates during a transient period of tumor development since established tumor cells (even those with a hypomethylated genome) generally display normal DNA methylation activities. Moreover, there is experimental evidence to suggest that CG gene hypomethylation in cancers results from a historical event of transient DNA demethylation [25].

By analyzing gene expression microarray data generated from a series of melanoma cell lines, we found that CG gene activation is correlated with the presence of a gene expression signature that has been previously associated with DNMT1 depletion. This expression signature was mainly characterized by the down-regulation of a set of genes (ICCG genes) showing enrichment for mitosis/division-related functions. In the present study, we investigated the possibility that CG gene activation in melanoma is the result of an episode of DNMT1 depletion. To this end, we compared the level of DNMT1 expression in melanoma tissue samples displaying either little or extensive activation of CG genes. Moreover, we developed cellular models, which allowed to confirm that transient depletion of DNMT1 can lead to coincident activation of CG genes and down-regulation of ICCG genes. Finally, the mechanisms involved in permanent down-regulation of ICCG genes were explored.

## Results

### A gene expression profile linking DNMT1 depletion with CG gene activation in melanoma cell lines

**Fig. 1:**
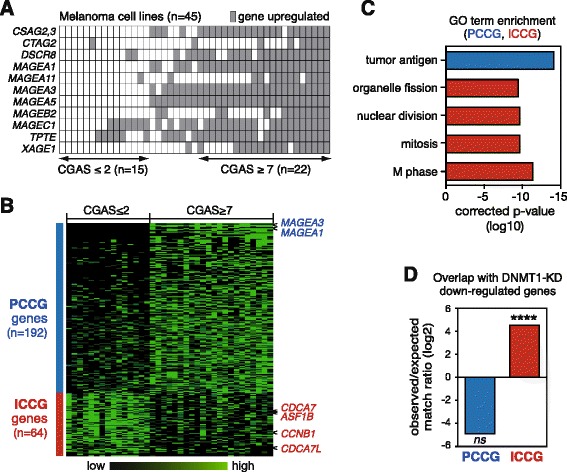
A gene expression profile links DNMT1 depletion with CG gene activation in melanoma cell lines. **a** Establishment of a CG gene activation score (CGAS) from the 45-MelCells dataset based on the expression profile of 11 selected CG genes (listed on the left). **b** Graphic representation of genes showing differential expression levels between the groups of melanoma cell lines displaying a CGAS either ≤2 or ≥7. The genes were classified from top to bottom according to their difference in mean expression. Two representative PCCG genes and four ICCG genes used in subsequent analyses are highlighted. **c** Functional analysis of PCCG and ICCG genes (via DAVID Bioinformatics Resources 6.7). Each bar in the histogram represents the Fisher exact *p* value (in a base 10 logarithmic scale) associated with the corresponding function in the group of genes. **d** Cross-comparison between the set of DNMT1-regulated genes (*n* = 346, see ref. [28]) and either the PCCG or ICCG group of genes. Overlap scores are expressed as ratio between observed and expected matches in a base 2 logarithmic (log2) scale. Statistical analysis was obtained by a Fisher test and compares in each group the number of observed genes vs the number of expected genes. ****p < 0.0001

In an effort to get better insight into the molecular pathways associated with the activation of CG genes in tumors, we undertook transcriptomic analyses aiming at identifying gene expression changes correlated with CG gene activation. To this end, we exploited a publicly available gene expression microarray dataset (GEO database GSE4843, hereafter designated 45-MelCells), which derives from a series of 45 human melanoma cell lines [26]. We chose this dataset because it is known that melanomas exhibit frequent activation of multiple CG genes [12] and because cell lines offer the advantage of a higher cellular homogeneity as compared to tumor tissues. A CG gene activation score (CGAS) was calculated for each of the 45 melanoma cell lines. This was performed by evaluating the number of activated CG genes among a set of 11 selected CG genes (Fig. 1a). CG genes in this reference set were selected because their probe intensity values were highly contrasted among the samples, thereby enabling clear distinction between their active or repressed state. CGAS values among melanoma cell lines did not show a normal distribution (D’Agostino and Pearson omnibus normality test: *p* value =0.0012) but rather indentified a group of 15 cell lines with no or only few activated CG genes (CGAS ≤2) and another group of 22 cell lines with a CGAS ≥7 (Fig. 1a). The 8 remaining cell lines displayed an intermediate CGAS, ranging between 4 and 6. Further analysis of the 45-MelCells dataset revealed that a majority of other tested CG genes also showed preferential activation in cell lines with a CGAS ≥7 (Additional file 1: Figure S1). Together, these observations confirmed previous data demonstrating that melanomas tend to display either no CG gene activation or coincident activation of multiple CG genes [27].

Microarray datasets were then further analyzed in order to identify genes showing differential expression levels between the two groups of melanoma cell lines displaying a CGAS either ≤2 or ≥7. Using a maximum 10 % false discovery rate and a minimum 2.0 difference of mean expression as criteria, only 14 genes were identified, which all showed increased expression in cell lines with a CGAS ≥7 (Additional file 2: Table S1). Not surprisingly, all these genes corresponded to previously characterized CG genes. This approach therefore did not allow us to identify genes, other than CG genes, displaying expression changes rigorously associated with CG gene activation. In particular, we found no evidence of association of CG gene activation with differential expression of genes involved in germline development.

Analysis of the 45-MelCells microarray dataset with less stringent statistical criteria (Mann–Whitney test, *p* value <0.03 and difference in mean expression ≥1.5) allowed identification of a larger set of genes that were differentially expressed according to the CGAS. Indeed, 192 genes, designated PCCG (*positively correlated* with *CG* gene activation), and 64 genes, termed ICCG (*inversely correlated* with *CG* gene activation), displayed a trend towards increased or decreased expression levels in melanoma cell lines with a CGAS ≥7 (Fig. 1b, Additional file 2: Table S1). Functional annotation analyses indicated that PCCG genes were enriched for the tumor antigen gene ontology term (Fig. 1c). This was not surprising considering that PCCG genes comprised many CG genes, in addition to the CG genes that were used to define the CGAS. Importantly, enrichment of CG genes among the PCCG group of genes supported the validity of the less stringent statistical approach. ICCG genes on the other hand, showed significant enrichment for mitosis/division-related gene ontology terms (Fig. 1c).

The observation that CG gene activation in melanoma cells is generally associated with down-regulation of genes involved in cell mitosis and division was rather unexpected. It was however reminiscent of a previous study by Sen and colleagues, who observed down-regulation of a set of cell mitosis/division-associated genes in epidermal cells that had been depleted of DNMT1 [28]. Interestingly, we found that the Sen set of DNMT1-regulated genes overlapped significantly with our group of ICCG genes (Fig. 1d, Additional file 3: Figure S2). Analysis of another study, where cells were exposed to a DNMT1 inhibitor (GSE30985), revealed similar down-regulation of cell mitosis/division-associated genes, as well as overlap of these genes with the ICCG group of genes (Additional file 4: Figure S3). These initial observations raised therefore the possibility that CG gene demethylation and activation in melanoma cells might be the consequence of a process of DNMT1 depletion.

### CG gene activation is associated with DNMT1 down-regulation in melanoma tissues

**Fig. 2:**
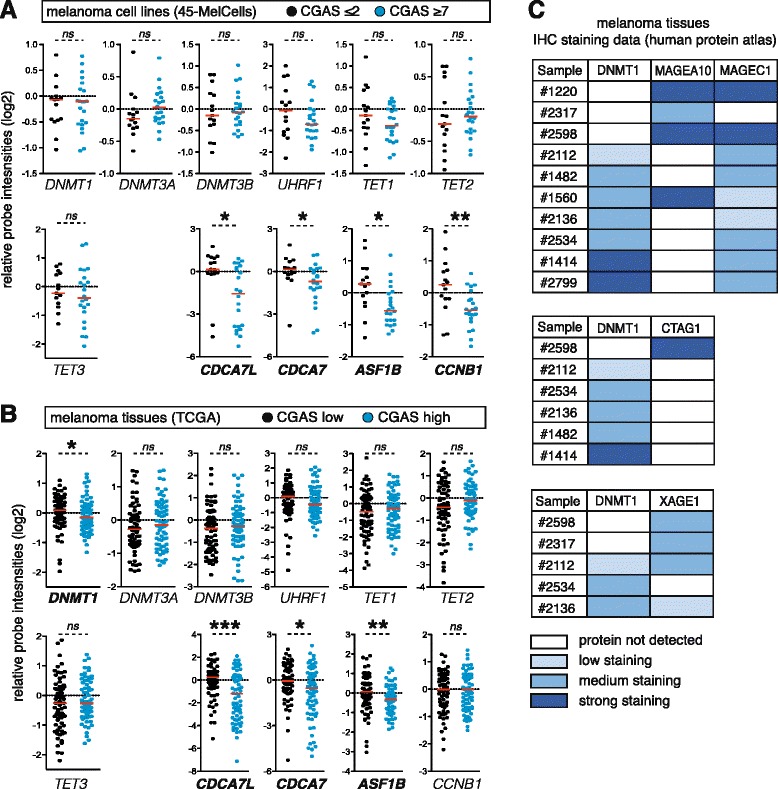
CG gene activation is associated with DNMT1 down-regulation in melanoma tissues. **a** The 45-MelCells dataset was analyzed for the expression of seven DNA methylation-associated genes (*DNMT1*, *DNMT3A*, *DNMT3B*, *UHRF1*, *TET1*, *TET2*, and *TET3*) and of the four representative ICCG genes. Graphs represent relative probe intensities for each gene (in a log2 scale), and each dot corresponds to one cell line. Cell lines were separated into two groups according to their CGAS (≤2 and ≥7). Differential gene expression between the two groups were evaluated (Mann–Whitney tests), and genes showing a significant *p* value are shown in bold (*p < 0.05, **p < 0.01). *Red bars* represent the median. **b** A similar analysis was performed in a dataset of melanoma tissue samples (TCGA). Samples were grouped according to their CGAS: CGAS-low and CGAS-high correspond to the lower and upper quartile, respectively (*n* = 70 in each group). *P* values were obtained as described here above. *p < 0.05, **p < 0.01, ***p < 0.001. **c** Analysis of immunohistochemistry (IHC)-based protein expression data in melanoma tissues (Human Protein Atlas). Melanoma samples where IHC data were available for both DNMT1 and CG protein levels are presented

If CG gene activation is associated with DNMT1 depletion in melanoma, one would expect to find an inverse correlation between the CGAS and the expression level of *DNMT1*. This was not the case in the 45 melanoma cell lines (Fig. 2a). However, when we analyzed an RNA-seq dataset obtained from a collection of 278 cutaneous melanoma tissue samples (TCGA), we observed that DNMT1 expression levels were slightly, albeit significantly, lower in samples displaying a higher CGAS (Fig. 2b; *p* = 0.0413), even when adjusting for cell proliferation rates (Additional file 5: Figure S4). Consistently, melanoma tissue samples with a higher CGAS also showed significant down-regulation of three out of out representative ICCG genes (*CDCA7L*, *CDCA7*, *ASF1B*, and *CCNB1*), which were selected from the original ICCG group on the basis of highest anti-correlation significance and variation coefficient in the 45-MelCells analysis (Fig. 2a, b). Further evidence for an association between DNMT1 down-regulation and CG gene activation in melanoma tissues was provided by the analysis of immunohistochemistry-based protein expression data (Human Protein Atlas), where we observed a trend towards weaker staining for DNMT1 in melanoma tissues scoring positive for CG proteins (Fig. 2c). Of note, the mRNA expression of other genes involved in DNA methylation (*DNMT3A*, *DNMT3B*, *UHRF1*, *TET1*, *TET2*, and *TET3*) showed no correlation with the CGAS in either melanoma cell lines or tissues (Fig. 2a, b). Moreover, analysis of clinical data from the TCGA collection of melanoma revealed no significant correlation between *DNMT1* expression levels and Clark staging or patient survival (Additional file 6: Figure S5).

Together our results indicate that CG gene activation in melanoma tissues is associated with reduced expression of DNMT1. Lack of such a correlation in melanoma cell lines may be related to the transient nature of the DNMT1 depletion process. Thus, while this depletion process is still in progress in several melanoma tissues, DNMT1 expression levels are likely restored in most melanoma cell lines that were expanded in vitro. CG gene activation and ICCG gene down-regulation would however persist past the transient phase of DNMT1 depletion. This scenario is in agreement with previous studies suggesting that CG gene activation in tumors results from a historical process of transient DNA demethylation [25, 29].

### Establishing cell systems for experimental depletion of DNMT1

**Fig. 3:**
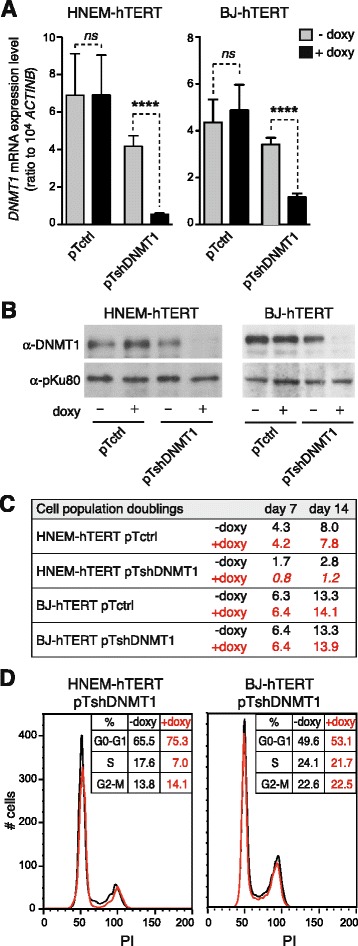
Validation of melanocyte and fibroblast cell systems for experimental depletion of DNMT1. **a** The *DNMT1* mRNA expression levels were measured by quantitative RT-PCR in HNEM-hTERT- and BJ-hTERT-derived pTctrl and pTshDNMT1 clones. The clones were exposed (+ doxy) or not (− doxy) to doxycycline (1 μg/mL—7 days). Expression values represent the mean (± sem) of at least three independent experiments. ****p < 0.0001. **b** Protein levels of DNMT1 and pKu80 (a loading control) were evaluated by Western-blot in the different clones, either with or without exposure to doxycycline. **c** The number of population doublings of HNEM-hTERT- and BJ-hTERT-derived clones, with or without 7 and 14 days doxycycline exposure, were determined by cell counting. Results derive from two independent experiments. **d** FACS analysis was performed on HNEM-hTERT- and BJ-hTERT-derived pT-shDNMT1 clones exposed or not to doxycycline (7 days). Propidium iodide staining (X axis) was measured and the percentage of cells in each cell cycle phase was determined

The results described above suggested that CG gene activation in melanoma results from a phase of DNMT1 depletion, which, at the same time, would induce down-regulation of ICCG genes. To further examine this possibility, we decided to verify if similar gene expression changes can be recapitulated following experimental depletion of DNMT1. To this end, we constructed a lentiviral vector harboring a doxycycline-inducible small hairpin RNA (shRNA) directed against DNMT1 (pTshDNMT1). The inducible transcript also comprised the sequence encoding the turbo Red Fluorescent protein (tRFP). A similar vector containing an irrelevant shRNA sequence was used as a control (pTctrl). The two vectors were transduced in immortalized human melanocytes (HNEM-hTERT) and fibroblasts (BJ-hTERT). Transduced clones carrying either the pTshDNMT1 or the pTctrl vector were isolated from these two cell types, and clones exhibiting near to 100 % tRFP positive cells after doxycycline exposure were selected (Additional file 7: Figure S6). RT-qPCR and immunoblotting analyses confirmed marked reduction of DNMT1 expression, at both the mRNA and protein level, in the pTshDNMT1 cell clones (but not in pTctrl clones) that had been exposed to doxycycline (Fig. 3a, b).

AS DNMT1 depletion has been shown to cause proliferation arrest in several cellular models [30, 31], we evaluated the effect of doxycycline exposure on the proliferation rate of both HNEM-hTERT and BJ-hTERT derived clones. Cell counting experiments and cell cycle analyses by flow cytometry indicated that whereas DNMT1 depletion markedly reduced proliferation of the HNEM-hTERT-derived pTshDNMT1 clone, it had only little impact on the growth of the BJ-hTERT-derived pTshDNMT1 clone (Fig. 3c, d).

### DNMT1 depletion leads to replication-dependent DNA demethylation and activation of CG genes

**Fig. 4:**
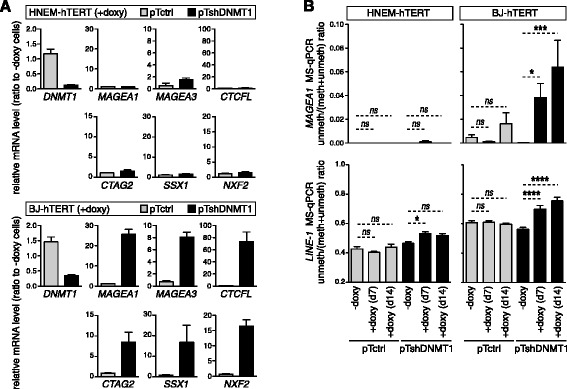
DNMT1 depletion leads to replication-dependent DNA demethylation and activation of CG genes. **a** The mRNA expression levels of *DNMT1* and of three CG genes (*MAGEA1*, *MAGEA3*, and *CTCFL*) were analyzed in HNEM-hTERT- and BJ-hTERT-derived clones exposed or not to doxycycline during 7 days. Values, which derive from al least three independent experiments, were normalized by the *ACTINB* expression level and are expressed relative to the levels found in − doxy cells. **b** DNA methylation levels within the *MAGEA1* 5'-region and *LINE-1* sequences were evaluated by MS-qPCR in the different clones, either with or without exposure (7 or 14 days) to doxycycline. Data represent the mean (± sem) of four independent qMS-PCR experiments, each in duplicate. *p < 0.05, ***p < 0.001, ****p < 0.0001

We next examined the activation of CG genes in HNEM-hTERT- and BJ-hTERT-derived pTshDNMT1 cell systems. Six CG genes (*MAGEA1*, *MAGEA3*, *CTCFL*, *CTAG2*, *SSX1*, *and NXF2*) were analyzed by RT-qPCR. The results indicated that whereas all six CG genes were markedly induced after 7 days of DNMT1 depletion in BJ-hTERT cells, their expression remained unchanged in similarly treated HNEM-hTERT cells (Fig. 4a). Consistently, analysis of the methylation status of the promoter of one of the CG genes (*MAGEA1*) by methylation-specific PCR revealed no apparent demethylation in the HNEM-hTERT-derived pTshDNMT1 cells that had been exposed for up to 14 days to doxycycline (Fig. 4b). In BJ-hTERT-derived pTshDNMT1 cells instead, DNMT1 depletion was accompanied by marked demethylation of the *MAGEA1* promoter (Fig. 4b). We also examined genome-wide DNA methylation changes, by using a methylation-specific PCR directed towards long interspersed elements (*LINE-1)*, which are found in large numbers across the human genome. The results indicated that whereas DNMT1 depletion-induced significant genome demethylation in BJ-hTERT cells, only little DNA methylation changes were observed in similarly treated HNEM-hTERT cells (Fig. 4b).

Together, our results validated the HNEM-hTERT and BJ-hTERT-derived clones as valuable systems to induce a phase of DNMT1 depletion. Consequent DNA demethylation and activation of CG genes was however observed only in BJ-hTERT-derived cells. Lack of a similar response in HNEM-hTERT cells is likely due to their proliferation arrest following depletion of DNMT1. Loss of DNA methylation upon DNMT1 depletion requires indeed several cycles of cell divisions.

### DNMT1 depletion induces down-regulation of ICCG genes

**Fig. 5:**
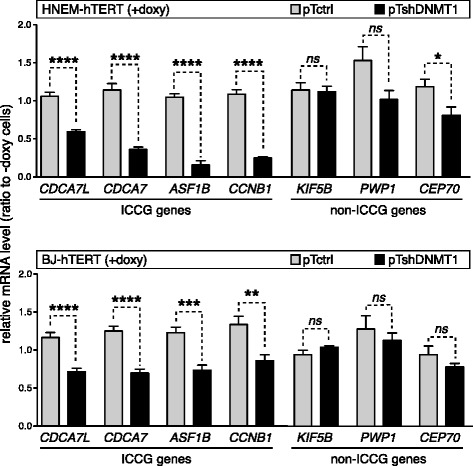
DNMT1 depletion induces down-regulation of ICCG genes. The mRNA expression levels of the four representative ICCG genes and of three non-ICCG genes (*KIF5B*, *PWP1*, and *CEP70*) were determined by RT-qPCR in HNEM-hTERT- and BJ-hTERT-derived clones with or without exposure to doxycycline (7 days). Values, which derived from at least four independent experiments, were normalized by the *ACTINB* expression level and are expressed relative to the levels found in − doxy cells

We then used the validated HNEM-hTERT- and BJ-hTERT-derived clones to investigate the effect of a phase of DNMT1 depletion on the expression of ICCG genes. To this end, pTshDNMT1 and pTctrl cell clones were maintained in doxycycline-containing or control medium during 7 days, and the mRNA level of the four reference ICCG genes (*CDCA7L*, *CDCA7*, *ASF1B*, and *CCNB1*) was analyzed by RT-qPCR. The results revealed significant decrease of all four ICCG mRNAs in doxycycline-treated pTshDNMT1 clones deriving from both HNEM-hTERT and BJ-hTERT cells (Fig. 5).

Considering that all four reference ICCG genes display cell cycle-associated expression (Additional file 8: Figure S7), it was possible that decreased mRNA amounts in DNMT1-depleted cells reflected a reduced proportion of proliferating cells rather than a process of gene repression. This was however unlikely to be the case for BJ-hTERT cells, which showed no obvious proliferation change upon DNMT1 depletion. To further confirm this, we tested the effect of DNMT1 depletion on the mRNA level of three genes (*KIF5B*, *PWP1*, and *CEP70*) that were not included in the ICCG group of genes but nevertheless display proliferation-associated expression, as evidenced by available cell cycle-associated mRNA expression data (http://www.cyclebase.org) and RT-qPCR experiments in serum-deprived BJ-hTERT cells (Additional file 8: Figure S7). The results showed that unlike ICCG genes, the three non-ICCG (yet proliferation-associated) genes displayed no significant mRNA decrease upon depletion of DNMT1 in BJ-hTERT cells (Fig. 5). In HNEM-hTERT cells, DNMT1 depletion was associated with reduced expression of *PWP1* and *CEP70*, probably as a consequence of the concurrent proliferation arrest in this cell type (Fig. 5).

Together, these observations confirm the association between depletion of DNMT1 and reduced expression of ICCG genes. Moreover, results in BJ-hTERT cells indicate that this association is not merely a consequence of reduced cell proliferation but likely involves a process of selective gene repression.

### Activation of CG genes and down-regulation of ICCG genes persist past a transient phase of DNMT1 depletion

**Fig. 6:**
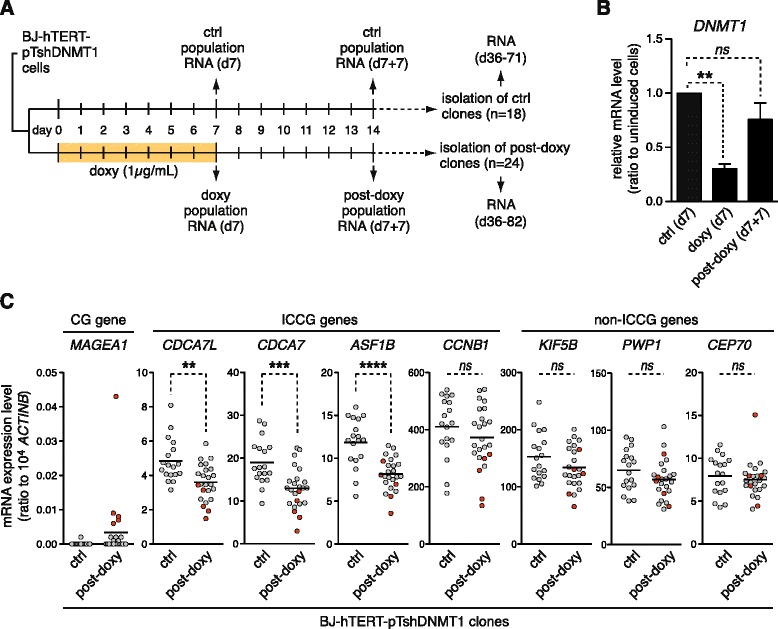
Activation of CG genes and down-regulation of ICCG genes persist past a transient phase of DNMT1 depletion. **a** Schematic outline of the experiment. BJ-hTERT-pTshDNMT1 cells were cultured in the absence (ctrl) or in the presence of doxycycline (doxy) for 7 days (d7), and thereafter, in normal medium during seven more days (d7 + 7). Control and post-doxy cell populations were then cloned by limiting dilution. RNA was extracted from clones between day 36 and 82. **b** *DNMT1* expression levels were analyzed by RT-qPCR in control and treated cell populations at the indicated time points. Data, which derived from at least four independent experiments, were normalized with *ACTINB* expression level and are expressed relative to the levels found in control cells. **p < 0.01. **c** Expression levels of the CG gene *MAGEA1*, the four ICCG genes, and the three non-ICCG genes were assessed by RT-qPCR in control and post-doxy clones. *Red dots* correspond to clones showing activation of *MAGEA1. Black bars* correspond to the mean. **p < 0.01, ***p < 0.001, ****p < 0.0001

As mentioned above, the phase of DNMT1 depletion at the origin of CG gene activation in tumors likely occurs transiently during tumor development. It was therefore essential to test if CG gene activation and ICCG gene down-regulation would persist past a temporary period of experimental depletion of DNMT1. We resorted exclusively to the BJ-hTERT-derived cell system for this analysis because HNEM-hTERT cells were found to cease proliferation upon DNMT1 depletion. Thus, BJ-hTERT-pTshDNMT1 cells were cultured in control or doxycycline-containing medium for 7 days. After this period, cells were transferred to normal medium and post-treatment clones were derived by limiting dilution (Fig. 6a). RT-qPCR experiments in treated cell populations confirmed that whereas the DNMT1 expression level was strongly reduced at the end of doxycycline exposure, it was almost completely restored 7 days after withdrawal of the drug (Fig. 6b). Clones deriving from either the control (18 clones) or doxycycline-treated (24 clones) BJ-hTERT-pTshDNMT1, cell populations were expanded in normal medium, and after up to 12 weeks, they were harvested for RT-qPCR analyses. In each of these clones, we evaluated the expression levels of a CG gene (*MAGEA1*), the four reference ICCG genes (*CDCA7L*, *CDCA7*, *ASF1B*, and *CCNB1*), and the three control non-ICCG genes (*KIF5B*, *PWP1*, and *CEP70*). RT-qPCR results for *MAGEA1* revealed persistent activation of this CG gene in 5 out of the 24 post-doxycycline clones and in none of the control clones (Fig. 6c). This suggested that only a proportion of post-doxycycline clones reached a sufficient threshold of DNMT1 depletion to permit CG gene demethylation and activation. RT-qPCR results for the ICCG genes revealed that 3 out of these genes (*CDCA7L*, *CDCA7*, and *ASF1B*) had expression levels that were significantly lower in post-doxycycline clones, as compared with control clones (Fig. 6c). None of the non-ICCG genes showed this trend (Fig. 6c). Interestingly, clones that showed *MAGEA1* activation displayed significantly lower levels of expression of all four ICCG genes, as compared with the other 19 post-doxycycline clones (Fig. 6c).

Together, these results confirm that depletion of DNMT1 can lead to coincident CG gene activation and ICCG gene down-regulation and indicate that these two opposite gene expression changes persist past the transient phase of DNMT1 depletion.

### RB1 is involved in ICCG gene regulation

**Fig. 7:**
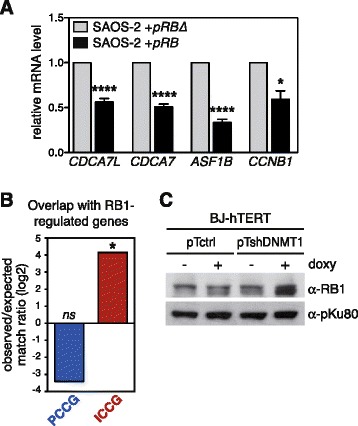
RB1 is involved in ICCG gene regulation. **a** mRNA expression levels of the four ICCG genes were evaluated by RT-qPCR in SAOS-2 cells transfected with a vector coding for the RB1 protein (*pRB*) or with a vector coding for a truncated and non-functional protein (*pRBΔ*). Data were normalized with *ACTINB* expression levels and are expressed relative to the levels found in the *pRBΔ* cells. Values represent the mean (± sem) of at least three independent experiments. *p < 0.05, ***p < 0.001. **b** Analysis for the presence of 165 previously characterized RB1-regulated genes [34] among PCCG and ICCG genes. Histograms represent the ratio between observed and expected matches, in a base 2 logarithmic scale. Statistical analysis was obtained by a Fisher test comparing observed vs. expected matches. *p < 0.05. **c** Western-blot was performed to analyze RB1 protein expression in BJ-hTERT-derived clones, either with or without 7 days exposure to doxycycline. pKu80 protein was used as a loading control

While we searched to identify factors possibly involved in transcriptional down-regulation of ICCG genes, we noticed that all four reference ICCG genes (*CDCA7L*, *CDCA7*, *ASF1B*, *CCNB1*) were previously reported to contain functional E2F binding elements within their promoter [32, 33]. This suggested that the E2F/RB1 pathway might be involved in the transcriptional regulation of these genes. To confirm this, we examined the level of mRNA expression of the four reference ICCG genes following restoration of RB1 expression in RB1-defective SAOS-2 cells. The results revealed significant down-regulation of all four ICCG genes in SAOS-2 cells that had been transfected with a RB1-encoding vector (Fig. 7a), suggesting that RB1 acts as a transcriptional repressor for these genes. In order to extend this finding to the other ICCG genes, we compared the list of 64 ICCG genes with a list of 165 previously identified RB1-regulated genes [34]. We observed significant overlap between these two lists of genes (Fig. 7b), thereby confirming enrichment of RB1-regulated genes within the group of ICCG genes. Moreover, analysis of gene expression data obtained from cells exposed to shRNA inhibitors directed against RB1 or against each of the two other RB-family proteins p107 and p130 suggested exclusive involvement of RB1 in the regulation of ICCG genes (Additional file 9: Figure S8). Interestingly, immunoblotting experiments revealed that DNMT1 depletion was associated with an increased amount of RB1 protein in BJ-hTERT cells (Fig. 7c). Together, these observations suggest that RB1 might contribute to the process of ICCG gene down-regulation that occurs during the phase of DNMT1 depletion.

### Epigenetic mechanisms associated with *CDCA7L* repression in melanoma cells

**Fig. 8:**
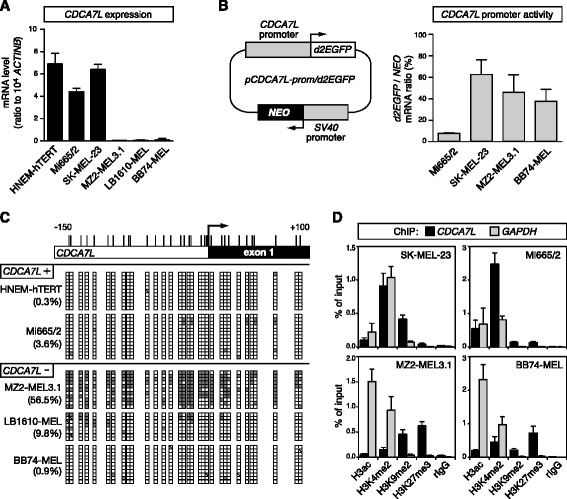
Epigenetic mechanisms associated with *CDCA7L* repression in melanoma cells. **a** *CDCA7L* mRNA expression levels were assessed in immortalized normal melanocytes (HNEM-hTERT) and in five melanoma cell lines (Mi665/2 and SK-MEL-23, MZ2-MEL3.1, LB1610-MEL, and BB74-MEL). Values represent mean (± sem) of at least two independent RT-qPCR experiments, each in duplicate. **b** Schematic representation of the *pCDCA7L-prom/d2EGFP* plasmid (*left panel*). Four melanoma cell lines were stably transfected with the *pCDCA7L-prom/d2EGFP* plasmid, and *d2EGFP* mRNA expression levels were evaluated (*right panel*). Results were normalized with *NEO* gene expression levels. Data represent mean (± sem) of three independent RT-qPCR analyses, each in duplicate. **c** Bisulfite sequencing analyses of the *CDCA7L* 5'-region were performed in HNEM-hTERT cells and in four melanoma cell lines displaying *CDCA7L* expression or repression (*CDCA7L*+ or −, respectively). Methylated and unmethylated CpGs are represented by *filled* and *empty boxes*, respectively. Overall CpG methylation percentages (%) are indicated. **d** Quantitative ChIP analyses were applied to four melanoma cell lines displaying either *CDCA7L* expression (SK-MEL-23, Mi665/2) or repression (MZ2-MEL3.1, BB74-MEL). Enrichment of indicated histone modifications were evaluated within the *CDCA7L* 5′-region (*GAPDH* served as a control, ubiquitously active, promoter). Data derive from at least two independent ChIP experiments, with two duplicate qPCR measures in each case

We next searched to get more insight into the molecular mechanisms that contribute to persistent down-regulation of ICCG genes in melanoma cells. To address this issue, we focused our studies on *CDCA7L* because repression of this ICCG gene was most significantly correlated with CG gene activation in melanoma tissues (Fig. 2b). Compared to other ICCG genes, *CDCA7L* also showed the most extensive level of repression in melanoma cell lines (Fig. 2a). This was confirmed by RT-qPCR experiments showing that whereas *CDCA7L* is expressed in melanocytes, it is almost completely silenced in several melanoma cell lines (Fig. 8a).

One possible explanation for the persistent repression of *CDCA7L* in melanoma cells was that DNMT1 depletion led to the irreversible activation of a factor that imposes silencing of the gene. To study this possibility, we decided to evaluate the activity of the *CDCA7L* promoter in melanoma cell lines that either do or do not express the gene. We reasoned that if *CDCA7L* repression is linked to the presence of a silencing factor, the promoter of the gene would show reduced activity in non-expressing melanoma cell lines. Thus, we constructed a plasmid vector in which the *CDCA7L* promoter was inserted upstream of the d2EGFP encoding sequence. The plasmid also contains a neomycin-resistance gene (*NEO*) under the control of the ubiquitously active SV40 promoter (Fig. 8b). This construct was stably transfected into two melanoma cell lines that express *CDCA7L* (Mi665/2 and SK-MEL-23) and two melanoma cell lines that show silencing of the gene (MZ2-MEL3.1 and BB74-MEL). Transfected cell populations were selected on the basis of neomycin resistance, and the *CDCA7L* promoter activity was evaluated by calculating the ratio between *d2EGFP* and *NEO* mRNA levels. The results revealed that the activity of the *CDCA7L* promoter was not significantly reduced in *CDCA7L*-negative cell lines, as compared with *CDCA7L*-positive cell lines (Fig. 8b). These observations argued against the involvement of a transcriptional repressor in the persistent repression of *CDCA7L* in melanoma cells.

Another possible explanation for the permanent repression of *CDCA7L* in melanoma cells was that down-regulation of this gene during the phase of DNMT1 depletion allowed local deposition of repressive epigenetic marks, which progressively locked the gene into an irreversible silent state. Such type of opportunistic process of epigenetic conversion has been previously described [25, 35]. We first examined if *CDCA7L* repression in melanoma cell lines was associated with DNA hypermethylation within its promoter. Bisulfite-sequencing experiments revealed that *CDCA7L* repression was associated with significant promoter hypermethylation in one cell line (MZ2-MEL3.1; Fig. 8c). However, two other *CDCA7L*-negative cell lines (LB1610-MEL and BB74-MEL) showed only little DNA methylation within the gene promoter (Fig. 8c), indicating that *CDCA7L* repression is not solely due to promoter hypermethylation. We therefore performed chromatin immunoprecipitation experiments to investigate if repression of the *CDCA7L* gene is associated with changes in histone modifications within its promoter region. The results indicated that *CDCA7L* repression was consistently associated with losses of the activating histone mark H3K4me2 and with gains in the repressive histone mark H3K27me3 (Fig. 8d). Altogether, these results support the hypothesis that permanent repression of *CDCA7L* in melanoma cells is linked to progressive deposition of repressive epigenetic marks within the gene promoter.

## Discussion

Because of the very restricted pattern of expression of CG genes and their frequent activation in a wide variety of tumors, antigens encoded by these genes are currently being tested in clinical trials of anti-cancer vaccination. Moreover, growing evidence indicates that several CG genes display oncogenic properties [13, 14], and it is expected that oncogenes with such a restricted pattern of expression will represent ideal targets for the development of anti-cancer therapies with limited side effects [36]. Understanding the epigenetic mechanisms that lead to CG gene activation in tumors constitutes an essential issue in these perspectives.

An intrinsic property of epigenetic modifications is that they remain in place even after the signal that initiated their establishment has dissipated. The molecular process at the origin of an epigenetic alteration can therefore be difficult to identify, as it may be no longer operating at the time of analysis. This probably explains why the mechanisms underlying CG gene demethylation and activation in tumors have remained unexplained. Our data indicate that, at least in melanoma, activation of CG genes is due to a phase of DNMT1 depletion. A first line of evidence was provided by our observation that activation of CG genes in melanoma cells correlates with the presence of a gene expression signature that has been previously associated with DNMT1 depletion [28]. This signature is characterized by the down-regulated expression of a set of mitosis/division-related genes (ICCG genes) and can therefore be related to the mitotic disturbances that were reported to occur upon DNMT1 depletion [30]. Our cellular models provided experimental confirmation of the impact of DNMT1 knockdown on ICCG gene expression. They also demonstrated that down-regulation of these genes persists past the phase of DNMT1 depletion. This explains why the gene expression signature could still be detected in melanoma cell lines, which otherwise showed restored DNMT1 expression levels. A second line of evidence implicating transient DNMT1 depletion as a causal factor of CG gene activation in melanoma was provided by careful examination of expression data deriving from a large set of melanoma tissues. This analysis revealed lower DNMT1 expression levels in melanoma samples showing activation of multiple CG genes. This suggests that part of the analyzed melanoma tissue samples were still undergoing the process of DNMT1 depletion at the time they were removed, and many of these samples also expressed CG genes. Together, our findings are consistent with previous in vitro studies demonstrating that experimental knockdown of DNMT1 constitutes a sufficient trigger to induce activation of multiple CG genes [37, 38]. Importantly, we now provide evidence that a process of DNMT1 depletion actually occurs in vivo in melanoma and is linked with CG gene activation.

Whereas we uncovered an epigenetically fixed gene expression signature in melanoma cells attesting their transition through an episode of DNMT1 depletion, no similar gene expression signature was observed in other tumor types. This suggests that DNA hypomethylation and CG gene activation rely on alternative mechanisms in other cancers, as evidenced by recent studies in brain and colon cancer [20, 21]. It remains possible, however, that tumors other than melanoma experience a phase of transient DNMT1 depletion, which in this case would not be associated with permanent acquisition of the gene expression signature we identified.

The down-regulated expression of mitosis/division-related genes during DNMT1 depletion likely reflects a cellular stress response. A stress response has been previously reported upon DNMT1 knockdown and was shown to evolve into mitotic catastrophe in the case of complete abolition of DNMT1 expression, for instance, following conditional deletion of the gene [30, 31]. The DNA damage sensing protein ATR appears to be involved in the DNMT1-depletion stress response [39], and our observations suggest contribution of the connected RB1 pathway. An intriguing observation is that down-regulation of mitosis/division-related genes is maintained past the phase of DNMT1 depletion. The physiological relevance of this phenomenon is unclear. With the exception of *CDCA7L*, affected mitosis/division-related genes displayed, however, only partial repression in melanoma cells. It is therefore likely that these genes retain a sufficient level of expression to support ongoing cell proliferation. On the long run, however, diminished expression of such genes may increase the rate of mitotic errors in tumor cells, and thereby, favor genomic instability.

The way the stress response induced by DNMT1 depletion impacts on cellular proliferation appears to vary according to the cell type. In our cellular models, we observed that DNMT1 depletion-induced cell cycle arrest in HNEM-hTERT melanocytes (essentially in G1) but not in BJ-hTERT fibroblasts. Our observation that the two cell types differed in the level of DNMT1 depletion that was reached upon induction of the anti-DNMT1 shRNA (66 % mRNA reduction in fibroblasts vs. 87 % in melanocytes) provides one possible explanation for these contrasting outcomes. Another explanation may relate to intrinsic differences between HNEM-hTERT melanocytes and BJ-hTERT fibroblasts in the molecular pathways that act downstream of the DNMT1 depletion-induced stress response. Importantly, whether cells do or do not arrest proliferation upon DNMT1 depletion determines the extent of subsequent DNA demethylation. Passive DNA demethylation resulting from lack of DNMT1 maintenance activity requires indeed several replication cycles. Consistently, we observed that DNMT1 depletion induced DNA hypomethylation and CG gene activation in unarrested fibroblasts but not in arrested melanocytes. A similar divergence in proliferative response to DNMT1 depletion may account for the observation that DNA methylation inhibitors induce CG gene activation more efficiently in tumor cells than in normal cells [40]. Clearly, understanding the molecular mechanisms that underlie such divergent proliferative reactions could help to predict cell type-specific propensities to undergo DNA demethylation upon DNMT1 inhibition. This may be critical when considering the use of DNMT1 inhibitors in anti-cancer therapies.

A major perspective will be to uncover the mechanisms that are responsible for the phase of DNMT1 depletion in melanoma cells. Several studies have reported DNA demethylation and reduced DNMT1 activities in cells approaching senescence [41–44]. Cellular senescence, which was shown to constitute an early (but escapable) barrier to melanoma development [45], represents therefore a possible origin to the phase of DNMT1 depletion. Tumor hypoxia represents another possible cause of DNMT1 depletion. DNA demethylation and decreased DNMT1 expression were indeed observed in cells that were cultured under hypoxic conditions [46–48]. As for many other tumor types, hypoxia represents an important step in melanoma progression [49].

## Conclusions

In summary, our present study provides in vivo evidence that aberrant activation of CG genes in melanoma is the result of a past event of DNMT1 depletion. This finding reveals therefore that activation of this group of germline-specific genes in tumor cells is due to a process of global disruption of DNA methylation activities rather than to awaking of a specific gametogenic program, as was previously proposed [11, 15]. An unexpected observation was that DNMT1 depletion leads not only to gene activation but also to irreversible down-regulation of a defined set of mitosis/division-related genes. This epigenetically fixed gene expression signature enabled us to track down the origin of CG gene activation in melanoma. An important observation of our study is therefore that gene expression signatures can be used to trace back the epigenetic history of tumors. This may proof valuable for tumor classification and, hence, therapeutic decision-making.

## Methods

### Analysis of melanoma cell lines and tissues datasets

For melanoma cell lines, we used the dataset GSE4843 (Mannheim dataset) from the GEO database, which was obtained on Affymetrix Human Genome U133 Plus 2.0 Array [26]. The raw signal intensity data had been previously scaled to an arbitrary mean value of 500 by MAS 5.0 software (Affymetrix). Genes with a score value inferior to 20 in all 45 cell lines were excluded from the analysis. The expression values of 11 CG genes (see Fig. 1a) in each sample was reported to the mean value in all samples. We used these relative expression levels to calculate the CG gene activation score (CGAS) for each cell line. The minimum threshold for CG gene activation was defined so as to match the proportion of CG gene negative/positive (~50 %) samples observed in human melanoma tumors [50]. To identify genes showing differential expression levels between melanoma cell lines with a CGAS either ≤2 or ≥7, we performed a nonparametric Mann–Whitney test and calculated the independent False Discovery Rate (FDR) for each gene in the dataset. In a first time, we used a maximum 10 % FDR and a minimum 2.0 difference of mean expression as selection criteria. Then, we resorted to a less stringent analysis by using the Mann–Whitney *p* value <0.03 and difference of mean expression ≥1.5 as selection thresholds. Functional analysis was performed using DAVID Bioinformatics Resource 6.7. For melanoma tissues, we resorted to RNA-seq datasets from the Skin Cutaneous Melanoma dataset (TCGA, provisional; *n* = 278), which accessed via the cBioPortal database [51, 52]. The CGAS was calculated as described above, and tissues were then separated into two groups: the “low CGAS” group, which corresponds to the lower quartile and is composed of 70 melanoma tissues expressing little CG genes, and the “high CGAS” group, which corresponds to the upper quartile and is composed of 70 melanoma tissues expressing multiple CG genes.

### Cell lines

All human melanoma cell lines, which derive from cutaneous melanoma metastases, were obtained from the Brussels Branch of the Ludwig Institute for Cancer Research and were cultured as previously described [9]. Immortalized human fibroblasts BJ-hTERT cells were kindly provided by F. d’Adda di Fagagna (IFOM foundation, Italy), and their culture conditions are described elsewhere [14]. Immortalized human melanocytes HNEM-hTERT cells were received from E. De Plaen (Ludwig Institute for Cancer Research, Belgium) and were cultured in MBM-4 medium supplemented with growth factors (#CC-3249, Lonza) and endothelin-3 (#CC-4510, Lonza). SAOS-2 osteosarcoma cell lines, which were a gift from F. Journe (ULB, Belgium), were cultured as previously described [53]. Cell cultures were maintained at 37 °C in a humidified atmosphere of 8 % CO_2_ for all melanoma cell lines and at 5 % CO_2_ for the other cell lines.

### Transductions and doxycycline treatment

The pTRIPZ-shDNMT1 (pTshDNMT1) vector was constructed by inserting a shRNA sequence directed against DNMT1 into the pTRIPZ-empty (pTctrl) vector (Thermo Scientific). The shRNA sequence was amplified from cells that were transduced with lentiviral particles coming from ThermoScientific Open Biosystems (#V3LHS_358136). To insert the shRNA sequence into the pTRIPZ vector, we resorted to primers 5′-CAGGTTAACCCAACAGAAGGCT-3′ and 5′-GTAATCCAGAGGTTGATTGTTCCA-3′, which carry a 5′-overhang containing a XhoI or a MluI restriction site, respectively. BJ-hTERT and HNEM-hTERT cells were then incubated with lentiviral supernatant containing either pTctrl or pTshDNMT1 plasmids and 8 μg/mL polybrene for 5 h. Three days later, cells were selected with 2.5 and 3 μg/mL puromycin (InvivoGen) for BJ-hTERT and HNEM-hTERT, respectively. Puromycin-resistant cells were finally cloned by limiting dilutions. To induce the shRNA expression from the pTshDNMT1 vector, stably transduced cells were submitted to the addition of 1 μg/mL of doxycycline (Clontech) into the culture medium for 7 or 14 days with renewal every second day.

### Plasmid constructions and transfections

The vector coding for the RB protein (plasmid 413 pSG5L HA RB from Addgene) was used to construct a control vector (*pRBΔ*). We used the EcoRI enzyme to digest the *pRB* vector into two fragments (of ~4.9 and 1.9 kb), and we kept the longer fragment to circularization. This control vector contains only the first tier of the total RB sequence and encodes a truncated and non-functional protein. The *pRB* or *pRBΔ* vector was transfected into SAOS-2 cells by using calcium phosphate coprecipitation. The culture medium was replaced 24 h after transfection by serum-free medium for an additional 24 h before RNA extraction.

To construct the *pCDCA7L-prom/d2EGFP* vector, we amplified a 2270 bp fragment of the *CDCA7L* promoter from human MZ2-MEL3.1 cells, using PrimeSTAR HS DNA Polymerase (Takara). Due to the presence of multiple HindIII restriction sites into the *CDCA7L*-promoter, a three sequential steps construction was performed. The first step was done to amplify the first part of the *CDCA7L* promoter (419 bp) by using primers 5′-CCGAAGCTTAGTATTACTGCAGTGCCATGT-3′ and 5′-AATTCAATCAGAGCTCTTCTTCCTCTTTT-3′, which carry a 5′-overhang HindIII and a SacI restriction site, respectively. This sequence was introduced into the *pMAGEA1/hph* vector [38], after digestion by HindIII and SacI, which at the same time allowed eviction of the *MAGEA1* sequence from the vector. Then, a second step was used to amplify the second part of the *CDCA7L* promoter (1851 bp) with the primers 5′-AAAAGAGGAAGAAGAGCTCTTGATTGAATT-3′ and 5′-GCCGTCGACTCTTCCTAACCGGGCTCCA-3′, which contain a SacI and a 5′-overhang SalI restriction site, respectively. This sequence was also introduced into the *pMAGEA1/hph* vector after digestion by SacI and SalI, immediately downstream of first part of the *CDCA7L* promoter sequence. Finally, a third step was performed to replace the *hph* coding sequence with the *d2EGFP* coding sequence. To this end, a fragment corresponding to the *d2EGFP* sequence was amplified from the *pCMV-d2EGFP-empty* plasmid (Addgene), by using the primers 5′-GGCGTCGACATGGTGAGCAAGGGCGAGGA-3′ and 5′-CGCGGCCGCCACATTGATCCTAGCAGAAGC-3′, which carry a 5′-overhang containing a SalI and a NotI restriction site, respectively. This PCR product was introduced instead of the *hph* sequence into the vector, now designated *pCDCA7L-prom/d2EGFP*. All constructs were verified by sequencing. For transfection of the *pCDCA7L-prom/d2EGFP* plasmid, we used the Lipofectamine 2000 reagent (Invitrogen) for MZ2-MEL3.1, BB74-MEL, and SK-MEL-23 cell lines and the TurboFect reagent (Thermo Scientific) for Mi665/2 cells, according to the manufacturer’s instructions. Transfections were performed in 25 cm^2^ flasks containing cells at about 90 % confluency in a medium without antibiotics. Cells were transfected with 10 μg of plasmid DNA, and the medium was replaced after 4 h of incubation. Two days after transfection, the cells were transferred into medium containing 1 mg/mL (MZ2-MEL3.1), 2 mg/mL (SK-MEL-23), 1.5 mg/mL (BB74-MEL), or 0.8 mg/mL (Mi665/2) of geneticin (Gibco, Life Technologies) during a minimum of 7 days. RNA was extracted between days 15 and 21 after transfection.

### Quantitative RT-PCR

Total RNA samples were extracted using TriPure Isolation Reagent (Roche Applied Science). Reverse transcription was generally performed on 2 μg of total RNA using oligo(dT) primers as described elsewhere [9]. Quantitative RT-PCR amplifications were performed by using the qPCR Core Kit (Eurogentec, Belgium). For qPCRs, either SybrGreen or Taqman assays were performed (see Additional file 10: Table S2). The primers and specific 5′-FAM/3′-TAMRA-labeled probes were synthesized commercially (Eurogentec), and their sequences are available in Additional file 10: Table S2. All qPCRs were performed in duplicates. Expression levels were generally normalized as a ratio to 10,000 *ACTINB* mRNA copies.

### Bisulfite genomic sequencing and quantitative MS-PCR

To analyze the methylation levels of the *MAGEA1* gene and *LINE-1* sequences, we resorted to quantitative methylation-specific PCR (qMS-PCR). The reaction conditions of the *MAGEA1* and *LINE-1* qMS-PCRs have been described elsewhere [53, 54]. To evaluate the relative levels of *MAGEA1* or *LINE-1* demethylation in our cell lines depleted or not in DNMT1, we calculated the ratio of unmethylated/(methylated + unmethylated) inferred from the -∂CTs in each sample. Methylation analyses of the *CDCA7L* promoter were performed by bisulfite sequencing as follows. A first PCR was performed on 0.25 μg of bisulfite-modified genomic DNA (50 s at 95 °C, 50 s at 60 °C, and 90 s at 72 °C for 35 cycles) with the following primers: 5′-TTTGYGAAGATAAGGTTAGTGGT-3′ (forward) and 5′-CCCACTACRCACACCTACAAA-3′ (reverse). Next, a second semi-nested PCR was performed on a dilution 1:150 of the first PCR products with the same amplification conditions. The forward primer was the same than for the first PCR, and the reverse primer was 5′-ACCRAACCCTCACCTAATAA-3′.

### FACS analysis on propidium iodide labeled cells

After 7 days of doxycycline exposure, cells were released by trypsin digestion, pelleted, and resuspended in 0.5 mL of 1× PBS. Then, 1.5 mL of cold 70 % ethanol was added dropwise to the cells, and the solution was stored at 4 °C. Prior flow cytometric analysis, the cells were pelleted and resuspended in 15 μL of 10 mg/ml RNAse. Two hundred microliter of 50 μg/ml propidium iodide diluted in 1× PBS was then added, and cells were incubated for 15 min at room temperature. Ten thousand single cell events per sample were analyzed for DNA content to identify G0/G1 (2N DNA content), G2/M (4N DNA content), and S-phase cells using a BD FACSVerse system (BD Biosciences). The FlowJo 9.8.2 software was used to analyze the data with the Watson Pragmatic model.

### Western-blotting

For Western-blotting against the RB protein, whole cell lysates were obtained by harvesting cells in RIPA lysis buffer complemented with cOmplete Mini Protease inhibitor cocktail (Roche) and PhosSTOP Phosphatase inhibitor cocktail (Roche). For all other Western-blottings, nuclear extracts were isolated via the NE-PER Nuclear and Cytoplasmic Extraction Reagents (Thermo Scientific) supplemented with cOmplete Mini Protease inhibitor cocktail (Roche). Whole cell lysates or nuclear extracts were denaturated for 10 min at 99 °C in the presence of Laemmli buffer 6× before loading and electrophoresis in a 8 % SDS-PAGE gel. Proteins were thereafter submitted to an electrotransfer on a polyvinylidene difluoride Immobilon®-P transfer membrane (Millipore) during 1 h and 30 mins at 100 V at 4 °C. The membrane was thereafter saturated in a PBS solution containing 5 % non-fat milk and 0.05 % Tween 20 during 1 h at room temperature. Incubation with the primary antibodies was performed in the same solution overnight at 4 °C for DNMT1, RB, or p80-Ku. Primary antibodies were as follows: anti-DNMT1 rabbit polyclonal antibody (1:2000, #ab19905, abcam), anti-Rb (C-15) rabbit polyclonal antibody (1:2000, #sc-50, Santa Cruz), and anti-p80-Ku mouse monoclonal antibody (1:1000, #05-393, Millipore). Following incubation with the primary antibody, the membrane was washed three times in PBS-Tween 0.05 % and then incubated at room temperature for 1 h in the presence of either HRP-conjugated goat anti-rabbit IgG antibody (1:10,000, #ADI-SAB-300, Enzo Life Sciences) or HRP-conjugated goat anti-mouse IgG antibody (1:2000, #sc-2005, Santa Cruz). Signals on the membrane were revealed using the SuperSignal® West Pico Chemiluminescent Substrate (Pierce, Thermo Scientific) and after exposure to Fuji Medical X-RAY films (Fujifilm). Before applying a new antibody, the membrane was subjected to a 10-min incubation in 0.4 M NaOH at room temperature, three washes in PBS-Tween 0.05 %, and incubation in a PBS solution containing 5 % non-fat milk and 0.05 % Tween 20 during 1 h at room temperature.

### ChIP assays and antibodies

Chromatin immunoprecipitation (ChIP) was performed on adherent cell lines at about 90 % confluency in a 150-mm culture dish containing 25 mL of growth media. ChIP assays were carried out by following the Upstate EZ-ChIP Kit protocol (Millipore, catalog no. 17–371), and chromatin was sheared with the Bioruptor Sonicator (Diagenode, cat. no. UCD-200 TM). The chromatin was immunoprecipitated using the following antibodies: anti-acetyl-Histone H3 polyclonal antibody (Upstate, cat. no. 06–599), anti-dimethyl-Histone H3 (Lys 4) polyclonal antibody (Active motif, cat. no. 39141), anti-dimethyl-Histone H3 (Lys 9) monoclonal antibody (Diagenode, cat. no. Mab-154-050), anti-trimethyl-Histone H3 (Lys 27) polyclonal antibody (Upstate, cat. no. 17–622), and normal rabbit IgG (Santa Cruz Biotechnology, cat. no. sc-2027). DNA purified from both the immunoprecipitated and pre-immune (input) samples was subjected to quantitative PCR amplification using the following primers and probes: 5′-CAAAGTGAACCCTGTAGCAA-3′(forward; −525), 5′-GCTGCAACCCCTGTCTCT-3′ (reverse; −389), 5′-6FAM-ACAAAACAAAACAAGCCCCAAAGCC-TAMRA-3′ (probe; −460) for the *CDCA7L* gene, and 5′-TACTAGCGGTTTTACGGGCG-3′ (forward; −230), 5′-CGAACAGGAGGAGCAGAGAGCGA-3′ (reverse; +46), 5′-6FAM-AGGCCTCAAGACCTTGGGCTGGGACTG-TAMRA-3′ (probe; −88) for the *GAPDH* gene. The results (% of input) were represented as the ratio of immunoprecipitated DNA/input DNA inferred from the -∂CTs in each sample.

## Additional files

Additional file 1: Figure S1. Activation profile of other CG genes in the 45-MelCells dataset. Relative expression values (ratio to mean in all samples) were obtained for all X-linked CG genes listed in Simpson et al. (*n* = 41; ref. [11]), but excluding the 11 reference CG genes used for determination of the CGAS. The threshold for activation was defined as described in the materials and methods section. A majority of CG genes (*n* = 26, shown in the upper part) shows preferential activation in melanoma cell lines with a CGAS ≥7. (PDF 3940 kb) Additional file 2: Table S1. List of 14 genes overexpressed in melanoma cell lines with a CGAS ≥7 (max 10 % FDR). List of 192 PCCG genes. List of 64 ICCG genes. (PDF 52 kb) Additional file 3: Figure S2. Overlap between ICCG genes and the set of genes that were down-regulated upon DNMT1 depletion in the Sen et al. study (ref. [28]). The Venn diagram shows exclusive overlap of the Sen set of genes with ICCG genes, but not with PCCG genes. The 21 genes common to ICCG genes and the Sen set of genes are listed. Reference ICCG genes, which were used in subsequent analyses are highlited in bold. The other reference ICCG gene, *CDCA7L,* is lacking in this list of common genes, because it was initially not represented on the microarray used by Sen et al. (PDF 3940 kb) Additional file 4: Figure S3. Exposure of HCT116 cells to DNMT1 inhibitor decitabine leads to down-regulation of cell mitosis/division-associated genes, and these genes show significant overlap with the group of ICCG genes. A) Genes showing transcriptonal up-regulation (fold change ≥4, adjusted *p* value ≤0.01, *n* = 165) or down-regulation (fold change ≤0.5, adjusted *p* value <0.01, *n* = 172) upon exposure to the DNMT1 inhibitor (GSE30985 dataset) were analyzed for enrichment of ontology terms. B) Overlap between down-regulated genes and ICCG genes was analyzed, and is expressed as observed/expected match ratio (log2). Fisher test was used for statiscal analysis: **p* ≤ 0.05. C) Changes of expression observed in cells treated with the DNMT1 inhibitor are given for the 11 reference CG genes, and for ICCG genes (those that overlap with the down-regulated group of genes in the Sen et al. study; see Additional file 3: Figure S2). (PDF 3940 kb) Additional file 5: Figure S4. Even after adjustment for cell proliferation, *DNMT1* mRNA expression levels remain lower in CGAS-high melanoma samples. As *DNMT1* shows a peak of mRNA expression during the S-phase of the cell cycle, reduced expression of this gene may reflect differences in cell proliferation rates rather than transcriptional down-regulation. To discriminate between these two possibilities, we resorted to a previously described strategy, wherein *DNMT1* expression levels were reported to that of another gene (*HIST2H4A*) showing peak expression during the S-phase (Lee et al. Proc Natl Acad Sci USA, 1996, 93:10366). A) Cell cycle-dependent mRNA expression levels, as provided by Cyclebase, confirm peak expression of *DNMT1* and *HIST2H4A* during the S-phase. B) DNMT1/HIST2H4A expression ratios were calculated (log2), and are represented in CGAS-low and CGAS-high melanoma samples (red bar at median). Statistical analysis was performed with the Mann–Whitney test. mRNA ratios remained lower in CGAS-high melanoma samples, thereby excluding differential proliferation rates as a cause of reduced *DNMT1* mRNA expression. (PDF 3940 kb) Additional file 6: Figure S5. DNMT1 expression levels show no correlation with histological staging or patient survival in cutaneous melanoma. DNMT1 mRNA expression levels and clinical data were obtained from the TCGA dataset of cutaneous melanoma. A) The graphs represents relative DNMT1 mRNA expression (log2 scale) in melanoma tissue samples grouped according to Clark stage (number of samples in each group is indicated between parentheses). Statistical analysis with Anova and Bonferroni’s multiple comparison tests revealed no significant differences. B) Melanoma samples were separated into DNMT1-low (<0.7 fold mean probe intensity level, *n* = 57) and DNMT1-high (>1,3 fold mean probe intensity level, *n* = 48), and a Kaplan-Meier plot was obtained for the two corresponding groups of patients. Log rank test revealed no significant difference in survival between the two groups. (PDF 3940 kb) Additional file 7: Figure S6. Illustration of selected pTctrl and pTshDNMT1 clones derived from either HNEM-hTERT or BJ-hTERT cells. Light and fluorescence microscopy images of selected clones, either without (− doxy) or after (+ doxy) treatment with 1 μg/mL of doxycycline during 3 days. Selected clones show ~100 % tTFP positive cells after doxycycline exposure. (PDF 3940 kb) Additional file 8: Figure S7. Evidence of cell cycle-associated expression of representative ICCG and non-ICCG genes. *A*, Cyclebase data of cell cycle mRNA expression peaktime for the 4 representative ICCG genes and the 3 non-ICCG genes (*KIF5B*, *PWP1* and *CEP70*). *B*, Relative mRNA levels of the genes were determined by RT-qPCR in BJ-hTERT fibroblasts cultured in the absence of serum during 7 days (− FBS d7). Values derived from 3 independent experiments and are expressed relative to the levels found in cells cultured in the presence of serum (+ FBS). ***p* <0.01, ****p* <0.001, *****p* <0.0001. (PDF 3940 kb) Additional file 9: Figure S8. shRNA-dependent inhibition of RB1, but not of p107 and p130, induces up-regulation of ICCG genes in human fibroblasts. Microarray expression data were extracted from the GSE19864 dataset (Chicas et al., Cancer Cell, 2010, 17:376). Fold change in expression is given for the 21 ICCG genes that overlapped with the Sen set of genes (see Additional file 3: Figure S2), and corresponds to the ratio (log2) of probe intensities between the indicated condition and control cells. (PDF 3940 kb) Additional file 10: Table S2. qPCR primers and probes. The sequences of PCR primers and Taqman probes (if used) are provided. (PDF 48 kb)
