# Supplementary material for: A gene expression signature identifying transient DNMT1 depletion as a causal factor of cancer-germline gene activation in melanoma
Source: Clin Epigenetics. 2015 Oct 26;7:114. doi: 10.1186/s13148-015-0147-4 (PMC4620642; doi:10.1186/s13148-015-0147-4)
Supplement: Additional file 1: Figure S1. — Activation profile of other CG genes in the 45-MelCells dataset. Relative expression values (ratio to mean in all samples) were obtained for all X-linked CG genes listed in Simpson et al. (n = 41; ref. [11]), but excluding the 11 reference CG genes used for determination of the CGAS. The threshold for activation was defined as described in the materials and methods section. A majority of CG genes (n = 26, shown in the upper part) shows preferential activation in melanoma cell lines with a CGAS ≥7. (PDF 3940 kb) [file 13148_2015_147_MOESM1_ESM.pdf]

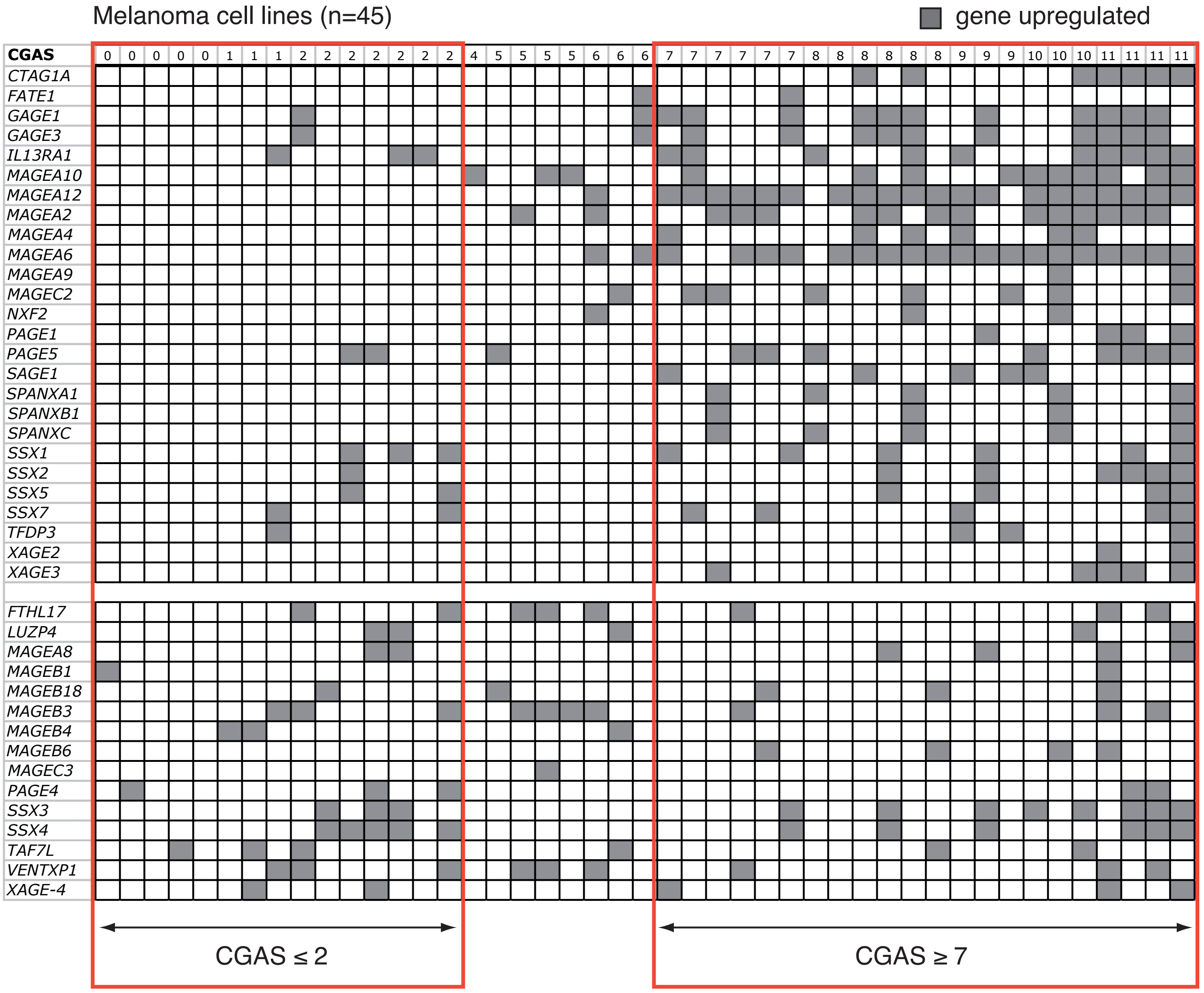

Figure S1. Activation profile of other CG genes in the 45-MelCells dataset. Relative expression values (ratio to mean in all samples) were obtained for all X-linked CG genes listed in Simpson et al. (n=41; ref. 11), but excluding the 11 reference CG genes used for determination of the CGAS. The threshold for activation was defined as described in the materials and methods section. A majority of CG genes (n=26, shown in the upper part) shows preferential activation in melanoma cell lines with a CGAS≥7.
