# Supplementary material for: A gene expression signature identifying transient DNMT1 depletion as a causal factor of cancer-germline gene activation in melanoma
Source: Clin Epigenetics. 2015 Oct 26;7:114. doi: 10.1186/s13148-015-0147-4 (PMC4620642; doi:10.1186/s13148-015-0147-4)
Supplement: Additional file 2: Table S1. — List of 14 genes overexpressed in melanoma cell lines with a CGAS ≥7 (max 10 % FDR). List of 192 PCCG genes. List of 64 ICCG genes. (PDF 52 kb) [file 13148_2015_147_MOESM2_ESM.pdf]

Table S1

List of 14 genes overexpressed in melanoma cell lines with a CGAS≥7 (max 10% FDR)

GeneNames	Mean in CGAS≤2	Mean in CGAS≥7	Fold diff	pval_wilcox	FDR.indep
MAGEA6	*82.62*	*5544.95*	*67.1*	*2.14E-10*	*2.16E-06*
MAGEA4	*21.96*	*1464.54*	*66.7*	*5.71E-06*	*1.69E-02*
MAGEA3	*103.52*	*5363.77*	*51.8*	*2.14E-10*	*2.16E-06*
MAGEA12	*72.45*	*3205.07*	*44.2*	*2.14E-10*	*2.16E-06*
MAGEA2	*38.42*	*1681.1*	*43.8*	*2.77E-06*	*1.01E-02*
MAGEA2B	*23.48*	*935.63*	*39.9*	*1.46E-07*	*8.19E-04*
MAGEA1	*57.22*	*1053.7*	*18.4*	*2.14E-10*	*2.16E-06*
MAGEA5	*69.6*	*1106.35*	*15.9*	*2.14E-10*	*2.16E-06*
MAGEB2	*23.94*	*370.81*	*15.5*	*1.86E-05*	*4.93E-02*
XAGE1A	*96.77*	*1483.34*	*15.3*	*2.97E-08*	*2.14E-04*
CSAG2	*44.65*	*663.59*	*14.9*	*1.50E-09*	*1.26E-05*
DSCR8	*73.24*	*759.71*	*10.4*	*5.71E-06*	*1.69E-02*
TPTE	*11.21*	*105.45*	*9.4*	*1.46E-07*	*8.19E-04*
MAGEA10	*80.56*	*234.41*	*2.9*	*3.40E-07*	*1.71E-03*

List of 192 PCCG genes

MAGEA6	*GAGE1*	*ISL1*
MAGEA4	*DNER*	*VCX2*
MAGEA3	*HAPLN1*	*RASEF*
MAGEA12	*MAGEC2*	*TM4SF18*
MAGEA2	*MDM2*	*LOC100129794*
CT45A1	*LOC643401*	*HNMT*
MAGEA1	*LOC51152*	*HTR2C*
MAGEA5	*PXDNL*	*PDE11A*
MAGEB2	*FAM133A*	*ODZ1*
XAGE1A	*LOC100289026*	*SPP1*
CSAG2	*LOC100509302*	*NCRNA00281*
CT45A1	*GABRG2*	*HRASLS*
MAGEA11	*CFI*	*PAGE1*
CTAG2	*LOC100507039*	*LOC100289550*
CTAG1A	*LOC401233*	*CPVL*
DSCR8	*RAB3IP*	*SLC25A21*
TPTE	*MAGEA10*	*LOC375295*
LOC100271840	*PCDHB14*	*MAL2*
MAGEC1	*CLCN4*	*ESRRG*
GAGE1	*ANGPT1*	*TXK*
GAGE3	*DEPDC6*	*C18orf2*
GAGE1	*LOC340340*	*CSMD1*
FSTL5	*GABRA2*	*SULF2*
LOC283352	*PCDHB3*	*LOC644450*
RAPGEF4	*CXorf48*	*AGT*
GAGE12C	*BAGE*	*PLK2*
LOC339535	*ABCC2*	*FLJ39632*
GAGE12F	*ZNF385D*	*CDKN1A*
NEFL	*NXT2*	*PTGFRN*
CYTL1	*HS6ST2*	*ICK*

OLIG2
TSPAN31
EPHX1
PITX2
RPL22L1
JAKMIP2
LOC646627
CCDC152
CCDC121
UXS1
LOC642236
HHAT
NEAT1
MAGEB6
FGF14
RRM2B
IMPAD1
LOC643783
GPCPD1
CDS1
CTTN
GABRA3
ABHD14A
LOC100506783
LOC100132288
MECOM
LOC100506691
LOC375010
CD55
ELOVL6
NBEA
ETNK1
AMACR
TIGD1
CDH6
PTPRG
IGF1R
LOC389834
TMEM106B
RNF180
TP53TG1
LOC100132288
KCNJ2
NOL3
MID1
ARSD
SPCS3
PIGM
PTGR2
SPRED1
PBX1
RNF213
ERO1L
RBM20
CHCHD7
PLAG1
NARG2
PHLDA1
TNFRSF10B
MDP1
TLCD1
C6orf138
SKAP2
SPPL2A
DIP2A
TTC30A
ZMAT3
RNASEL
LOC100505490
RPS27L
ZMYM6
SERPINA1
LEPREL4
NPL
LMBRD2
KIAA1324L
SEPP1
NLGN4X
DUSP19
C18orf32
TNPO1
LOC100216479
EPT1
TPK1
SRD5A3
CENPBD1
MSI2
AKT3
SLC48A1
GALNT7
SLC7A11
APOOL
MAN1A2
ORMDL2
C16orf52
TRIM69
SLC16A4
TRAM1L1
SBNO1
LRPAP1
LOC100129361
NAPRT1

List of 64 ICCG genes

ACAT2
ANGPT2
ASF1B
ASPM
C12orf48
C6orf174
CCDC18
CCNA2
CCNB1
CDC20
CDCA7
CDCA7L
CDK1
CDKN2A
CELF2
CKS2
CSTF3
DIAPH3
DSC3
FAM201A
FSD1L
HAUS6
HELLS
HNRNPA3
HNRPDL
ITGAL
KIF11
KIF15
KIF2C
LBH
LHX2
LMO4
LPAR3
MICB
MKI67
MTF2
N4BP2L1
NAA38
NAV1
NET1
NUDT5
PDZRN3
PHF19
PLK4
RAD51AP1
RBM17
RNASEH2B
RUNX3

SAMD5	*TAF5*	*VRK1*
SCAI	*TFDP2*	*WNT4*
SENP6	*TIPRL*	*ZNF738*
SH2D1A	*TRIM59*	*ZNF823*
SMC4	*TTK*	
SMC6	*UB3*	
