# Supplementary material for: A gene expression signature identifying transient DNMT1 depletion as a causal factor of cancer-germline gene activation in melanoma
Source: Clin Epigenetics. 2015 Oct 26;7:114. doi: 10.1186/s13148-015-0147-4 (PMC4620642; doi:10.1186/s13148-015-0147-4)
Supplement: Additional file 3: Figure S2. — Overlap between ICCG genes and the set of genes that were down-regulated upon DNMT1 depletion in the Sen et al. study (ref. [28]). The Venn diagram shows exclusive overlap of the Sen set of genes with ICCG genes, but not with PCCG genes. The 21 genes common to ICCG genes and the Sen set of genes are listed. Reference ICCG genes, which were used in subsequent analyses are highlited in bold. The other reference ICCG gene, CDCA7L, is lacking in this list of common genes, because it was initially not represented on the microarray used by Sen et al. (PDF 3940 kb) [file 13148_2015_147_MOESM3_ESM.pdf]

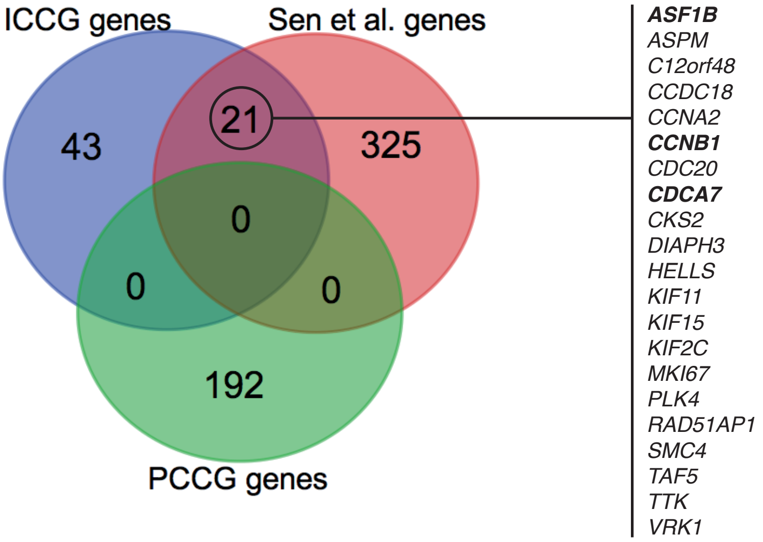

Figure S2. Overlap between ICCG genes and the set of genes that were down-regulated upon DNMT1 depletion in the Sen et al. study (ref. 28). The Venn diagram shows exclusive overlap of the Sen set of genes with ICCG genes, but not with PCCG genes. The 21 genes common to ICCG genes and the Sen set of genes are listed. Reference ICCG genes, which were used in subsequent analyses are highlited in bold. The other reference ICCG gene, *CDCA7L,* is lacking in this list of common genes, because it was initially not represented on the microarray used by Sen et al.
