# Supplementary material for: A gene expression signature identifying transient DNMT1 depletion as a causal factor of cancer-germline gene activation in melanoma
Source: Clin Epigenetics. 2015 Oct 26;7:114. doi: 10.1186/s13148-015-0147-4 (PMC4620642; doi:10.1186/s13148-015-0147-4)
Supplement: Additional file 4: Figure S3. — Exposure of HCT116 cells to DNMT1 inhibitor decitabine leads to down-regulation of cell mitosis/division-associated genes, and these genes show significant overlap with the group of ICCG genes. A) Genes showing transcriptonal up-regulation (fold change ≥4, adjusted p value ≤0.01, n = 165) or down-regulation (fold change ≤0.5, adjusted p value <0.01, n = 172) upon exposure to the DNMT1 inhibitor (GSE30985 dataset) were analyzed for enrichment of ontology terms. B) Overlap between down-regulated genes and ICCG genes was analyzed, and is expressed as observed/expected match ratio (log2). Fisher test was used for statiscal analysis: *p ≤ 0.05. C) Changes of expression observed in cells treated with the DNMT1 inhibitor are given for the 11 reference CG genes, and for ICCG genes (those that overlap with the down-regulated group of genes in the Sen et al. study; see Additional file 3: Figure S2). (PDF 3940 kb) [file 13148_2015_147_MOESM4_ESM.pdf]

A

B

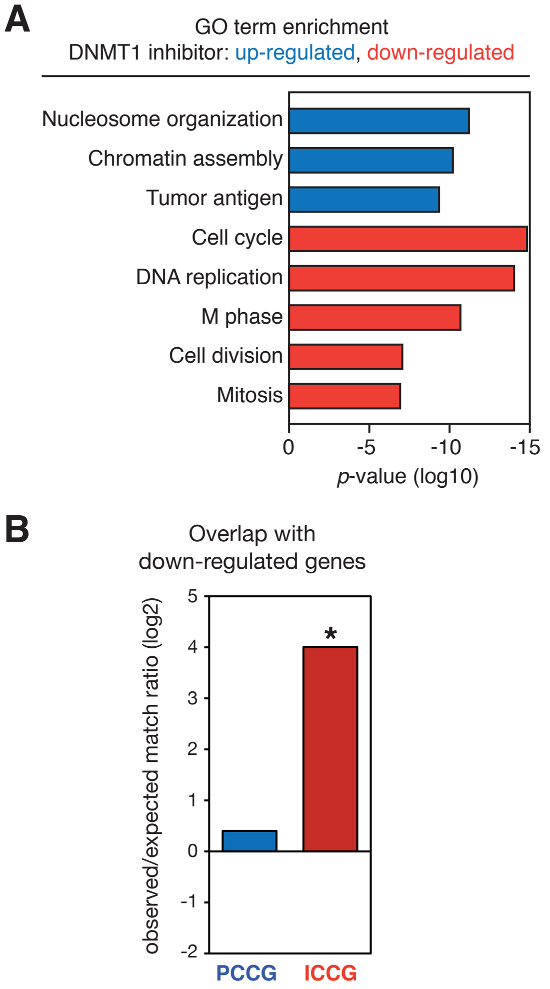

C

Gene expression changes associated with exposure to a DNMT1 inhibitor

CG genes	Fold change	p-value	ICCG genes	Fold change	p-value
XAGE1B	18,25	5,4E-12	*KIF15*	0,26	2,4E-08
MAGEA1	13,27	2,1E-10	*CDCA7*	0,33	5,5E-08
MAGEA11	8,11	1,9E-09	*KIF11*	0,38	2,8E-05
CTAG2	5,90	2,8E-10	*MKI67*	0,39	7,4E-07
MAGEA5	1,97	5,2E-03	*SMC4*	0,42	4,3E-07
MAGEA3	1,43	2,0E-04	*ASPM*	0,44	7,7E-06
MAGEB2	1,40	6,2E-04	*C12orf48*	0,47	1,3E-05
CSAG3	1,38	2,7E-03	*HELLS*	0,49	8,6E-06
MAGEC1	1,38	2,1E-03	*CCDC18*	0,51	5,3E-06
TPTE	1,06	5,9E-01	*PLK4*	0,52	4,0E-06
DSCR8	0,97	7,3E-01	*TTK*	0,54	6,9E-05
			CCNA2	0,58	8,2E-06
			CDCA7L	0,58	7,8E-06
			ASF1B	0,59	1,7E-05
			VRK1	0,59	2,7E-04
			KIF2C	0,66	6,0E-05
			DIAPH3	0,69	3,4E-04
			RAD51AP1	0,71	5,6E-02
			CDC20	0,73	7,0E-04
			TAF5	0,76	5,1E-03
			CCNB1	0,83	4,3E-02
			CKS2	1,00	9,7E-01

Figure S3. Exposure of HCT116 cells to DNMT1 inhibitor decitabine leads to down-regulation of cell mitosis/division-associated genes, and these genes show significant overlap with the group of ICCG genes. A) Genes showing transcriptonal up-regulation (fold change ≥ 4, adjusted *p*-value ≤ 0.01, n=165) or down-regulation (fold change ≤ 0.5, adjusted p-value < 0.01, n=172) upon exposure to the DNMT1 inhibitor (GSE30985 dataset) were analyzed for enrichment of ontology terms. **B)** Overlap between down-regulated genes and ICCG genes was analyzed, and is expressed as observed/expected match ratio (log2). Fisher test was used for statiscal analysis: * $p \leq 0.05$. **C)** Changes of expression observed in cells treated with the DNMT1 inhibitor are given for the 11 reference CG genes, and for ICCG genes (those that overlap with the down-regulated group of genes in the Sen et al. study; see Fig. S2).
