# Supplementary material for: A gene expression signature identifying transient DNMT1 depletion as a causal factor of cancer-germline gene activation in melanoma
Source: Clin Epigenetics. 2015 Oct 26;7:114. doi: 10.1186/s13148-015-0147-4 (PMC4620642; doi:10.1186/s13148-015-0147-4)
Supplement: Additional file 5: Figure S4. — Even after adjustment for cell proliferation, DNMT1 mRNA expression levels remain lower in CGAS-high melanoma samples. As DNMT1 shows a peak of mRNA expression during the S-phase of the cell cycle, reduced expression of this gene may reflect differences in cell proliferation rates rather than transcriptional down-regulation. To discriminate between these two possibilities, we resorted to a previously described strategy, wherein DNMT1 expression levels were reported to that of another gene (HIST2H4A) showing peak expression during the S-phase (Lee et al. Proc Natl Acad Sci USA, 1996, 93:10366). A) Cell cycle-dependent mRNA expression levels, as provided by Cyclebase, confirm peak expression of DNMT1 and HIST2H4A during the S-phase. B) DNMT1/HIST2H4A expression ratios were calculated (log2), and are represented in CGAS-low and CGAS-high melanoma samples (red bar at median). Statistical analysis was performed with the Mann–Whitney test. mRNA ratios remained lower in CGAS-high melanoma samples, thereby excluding differential proliferation rates as a cause of reduced DNMT1 mRNA expression. (PDF 3940 kb) [file 13148_2015_147_MOESM5_ESM.pdf]

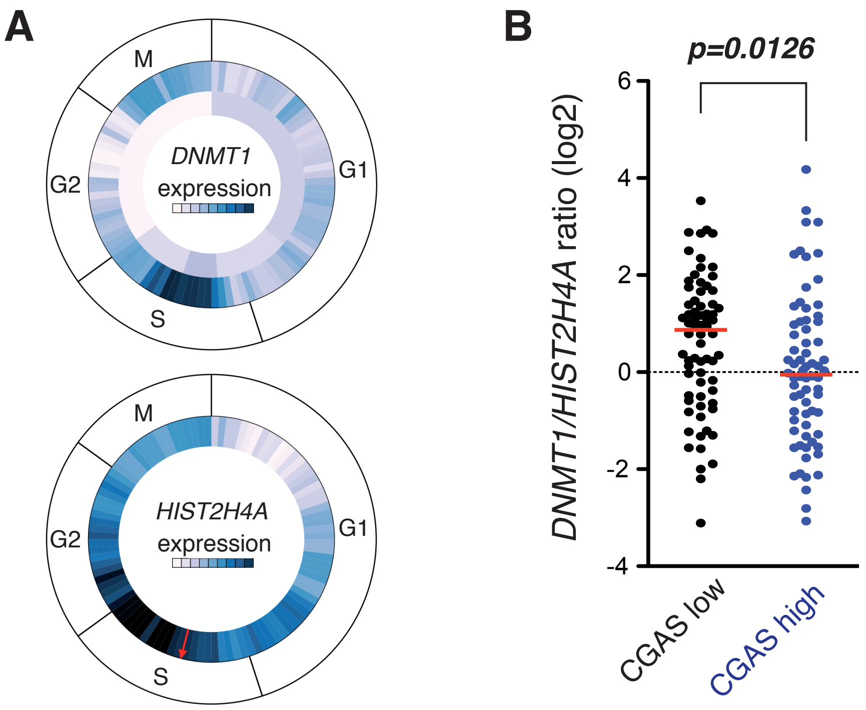

Figure S4. Even after adjustment for cell proliferation, *DNMT1* mRNA expression levels remain lower in CGAS-high melanoma samples. As *DNMT1* shows a peak of mRNA expression during the S phase of the cell cycle, reduced expression of this gene may reflect differences in cell proliferation rates rather than transcriptional down-regulation. To discriminate between these two possibilities, we resorted to a previously described strategy, wherein *DNMT1* expression levels were reported to that of another gene (*HIST2H4A*) showing peak expression during the S-phase (Lee et al. Proc Natl Acad Sci USA, 1996, 93:10366). **A)** Cell cycle-dependent mRNA expression levels, as provided by Cyclebase, confirm peak expression of *DNMT1* and *HIST2H4A* during the S phase. **B)** DNMT1/HIST2H4A expression ratios were calculated (log2), and are represented in CGAS-low and CGAS-high melanoma samples (red bar at median). Statistical analysis was performed with the Mann-Whitney test. mRNA ratios remained lower in CGAS-high melanoma samples, thereby excluding differential proliferation rates as a cause of reduced *DNMT1* mRNA expression.
