# Supplementary material for: A gene expression signature identifying transient DNMT1 depletion as a causal factor of cancer-germline gene activation in melanoma
Source: Clin Epigenetics. 2015 Oct 26;7:114. doi: 10.1186/s13148-015-0147-4 (PMC4620642; doi:10.1186/s13148-015-0147-4)
Supplement: Additional file 6: Figure S5. — DNMT1 expression levels show no correlation with histological staging or patient survival in cutaneous melanoma. DNMT1 mRNA expression levels and clinical data were obtained from the TCGA dataset of cutaneous melanoma. A) The graphs represents relative DNMT1 mRNA expression (log2 scale) in melanoma tissue samples grouped according to Clark stage (number of samples in each group is indicated between parentheses). Statistical analysis with Anova and Bonferroni’s multiple comparison tests revealed no significant differences. B) Melanoma samples were separated into DNMT1-low (<0.7 fold mean probe intensity level, n = 57) and DNMT1-high (>1,3 fold mean probe intensity level, n = 48), and a Kaplan-Meier plot was obtained for the two corresponding groups of patients. Log rank test revealed no significant difference in survival between the two groups. (PDF 3940 kb) [file 13148_2015_147_MOESM6_ESM.pdf]

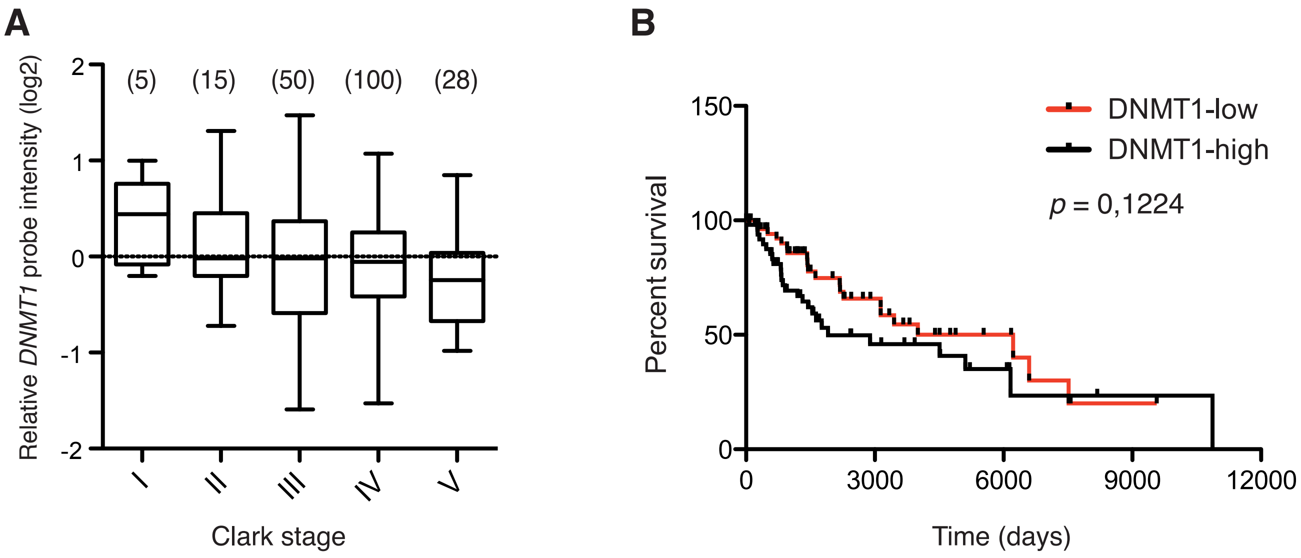

Figure S5. DNMT1 expression levels show no correlation with histological staging or patient survival in cutaneous melanoma. DNMT1 mRNA expression levels and clinical data were obtained from the TCGA dataset of cutaneous melanoma. **A)** The graphs represents relative DNMT1 mRNA expression (log2 scale) in melanoma tissue samples grouped according to Clark stage (number of samples in each group is indicated between parentheses). Statistical analysis with Anova and Bonferroni's multiple comparison tests revealed no significant differences. **B)** Melanoma samples were separated into DNMT1-low (<0.7 fold mean probe intensity level, n=57) and DNMT1-high (>1,3 fold mean probe intensity level, n=48), and a Kaplan-Meier plot was obtained for the two corresponding groups of patients. Log rank test revealed no significant difference in survival between the two groups.
