# Supplementary material for: A gene expression signature identifying transient DNMT1 depletion as a causal factor of cancer-germline gene activation in melanoma
Source: Clin Epigenetics. 2015 Oct 26;7:114. doi: 10.1186/s13148-015-0147-4 (PMC4620642; doi:10.1186/s13148-015-0147-4)
Supplement: Additional file 7: Figure S6. — Illustration of selected pTctrl and pTshDNMT1 clones derived from either HNEM-hTERT or BJ-hTERT cells. Light and fluorescence microscopy images of selected clones, either without (− doxy) or after (+ doxy) treatment with 1 μg/mL of doxycycline during 3 days. Selected clones show ~100 % tTFP positive cells after doxycycline exposure. (PDF 3940 kb) [file 13148_2015_147_MOESM7_ESM.pdf]

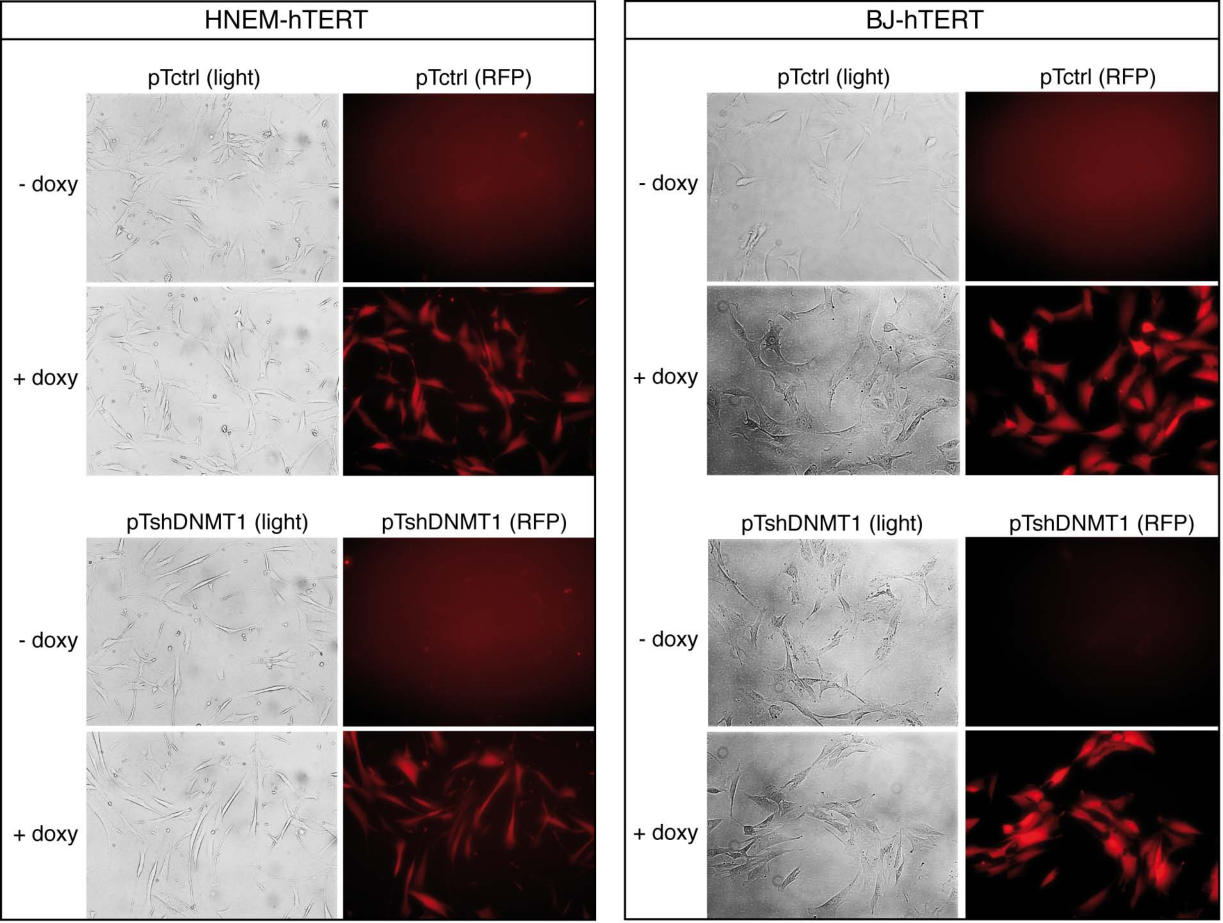

Figure S6. Illustration of selected pTctrl and pTshDNMT1 clones derived from either HNEM-hTERT or BJ-hTERT cells. Light and fluorescence microscopy images of selected clones, either without (- doxy) or after (+ doxy) treatment with 1 µg/mL of doxycycline during 3 days. Selected clones show ~100% tRFP positive cells after doxycycline exposure.
