# Supplementary material for: A gene expression signature identifying transient DNMT1 depletion as a causal factor of cancer-germline gene activation in melanoma
Source: Clin Epigenetics. 2015 Oct 26;7:114. doi: 10.1186/s13148-015-0147-4 (PMC4620642; doi:10.1186/s13148-015-0147-4)
Supplement: Additional file 8: Figure S7. — Evidence of cell cycle-associated expression of representative ICCG and non-ICCG genes. A, Cyclebase data of cell cycle mRNA expression peaktime for the 4 representative ICCG genes and the 3 non-ICCG genes (KIF5B, PWP1 and CEP70). B, Relative mRNA levels of the genes were determined by RT-qPCR in BJ-hTERT fibroblasts cultured in the absence of serum during 7 days (− FBS d7). Values derived from 3 independent experiments and are expressed relative to the levels found in cells cultured in the presence of serum (+ FBS). **p <0.01, ***p <0.001, ****p <0.0001. (PDF 3940 kb) [file 13148_2015_147_MOESM8_ESM.pdf]

A

Cyclebase data	
Gene name	mRNA peaktime
CDCA7L	G1/S
CDCA7	G1
ASF1B	S
CCNB1	M
KIF5B	M
PWP1	M
CEP70	M

B

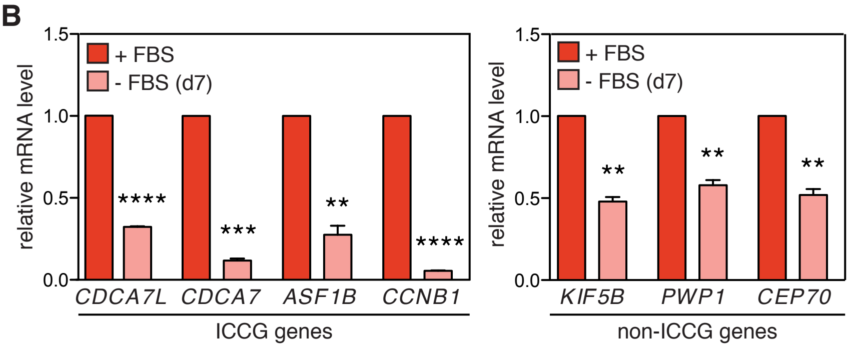

Figure S7. Evidence of cell cycle-associated expression of representative ICCG and non-ICCG genes. *A*, Cyclebase data of cell-cycle mRNA expression peaktime for the 4 representative ICCG genes and the 3 non-ICCG genes (*KIF5B*, *PWP1* and *CEP70*). *B*, Relative mRNA levels of the genes were determined by RT-qPCR in BJ-hTERT fibroblasts cultured in the absence of serum during 7 days (- FBS d7). Values derived from 3 independent experiments and are expressed relative to the levels found in cells cultured in the presence of serum (+ FBS). **p<0.01, ***p<0.001, ****p<0.0001.
