# Supplementary material for: A gene expression signature identifying transient DNMT1 depletion as a causal factor of cancer-germline gene activation in melanoma
Source: Clin Epigenetics. 2015 Oct 26;7:114. doi: 10.1186/s13148-015-0147-4 (PMC4620642; doi:10.1186/s13148-015-0147-4)
Supplement: Additional file 9: Figure S8. — shRNA-dependent inhibition of RB1, but not of p107 and p130, induces up-regulation of ICCG genes in human fibroblasts. Microarray expression data were extracted from the GSE19864 dataset (Chicas et al., Cancer Cell, 2010, 17:376). Fold change in expression is given for the 21 ICCG genes that overlapped with the Sen set of genes (see Additional file 3: Figure S2), and corresponds to the ratio (log2) of probe intensities between the indicated condition and control cells. (PDF 3940 kb) [file 13148_2015_147_MOESM9_ESM.pdf]

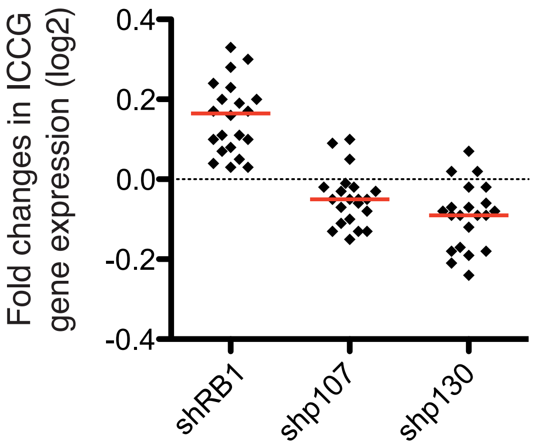

Figure S8. shRNA-dependent inhibition of RB1, but not of p107 and p130, induces up-regulation of ICCG genes in human fibroblasts. Microarray expression data were extracted from the GSE19864 dataset (Chicas et al., Cancer Cell, 2010, 17:376). Fold change in expression is given for the 21 ICCG genes that overlapped with the Sen set of genes (see Fig. S2), and corresponds to the ratio (log2) of probe intensities between the indicated condition and control cells.
