# Supplementary material for: A gene expression signature identifying transient DNMT1 depletion as a causal factor of cancer-germline gene activation in melanoma
Source: Clin Epigenetics. 2015 Oct 26;7:114. doi: 10.1186/s13148-015-0147-4 (PMC4620642; doi:10.1186/s13148-015-0147-4)
Supplement: Additional file 10: Table S2. — qPCR primers and probes. The sequences of PCR primers and Taqman probes (if used) are provided. (PDF 48 kb) [file 13148_2015_147_MOESM10_ESM.pdf]

Table S2. qPCR primers and probes

Target gene	Forward sequence 5' to 3'	Reverse sequence 5' to 3'	Probe sequence 5' to 3'
ACTINB	CCCTGGACTTCGAGCAAGAGAT	AAGGTAGTTTCGTGGATGCCACA	SybrGreen
DNMT1	CCTCAGCTACTGCGACTACT	GCACTCATGTCCTTACAGATGT	ATGGTCCTGAAGCTCACCCTCC
MAGEA1	GCCGAAGGAACCTGACC	ACTGGGTTGCCTCTGTCG	TGTGTGCAGGCTGCCACCTCCT
MAGEA3	GTCGTCGGAAATTGGCAGTAT	GCAGGTGGCAAAGATGTACAA	AAAGCTTCCAGTTCCTT
CTCFL	CCCTCATTCAGCACCAGAA	CTGATCCACACTTCTCCGAA	CCGACAGAAGCAACTTCTAAACGCT
CDCA7L	CTATGGGGAGGATGTCAGAT	GGTGGCTGGTTCTGTTTGT	TGTTAAGGAATATCTGGAGAGCTTACAAAA
CDCA7	CACTGGGCTCTACTTGTCATC	GCAGATTCCTCGACAAGGCG	SybrGreen
ASF1B	GGACGACCTGGAGTGGAAGAT	CATCAGTCTCTGGGATGAGGGA	SybrGreen
CCNB1	CATGCAGAATAATTGTGTGCCC	AGGAAGTGCAAAGGTAGAGGC	SybrGreen
PWP1	AAGGGCTTCGGGTCTGGGATA	GTGGTGCCACCGCAGTTGCA	SybrGreen
KIF5B	GCTTGATGACAAGGATGAAGAA	GCTTGAAGGCGATTCAGCTCA	SybrGreen
CEP70	TCAGGTGCTGTGTAGCATCAA	GCCAAGGTACCAGTTCTGCAGATA	SybrGreen
d2EGFP	ACGACGGCAACTACAAGACC	CTCAGGTAGTGGTTGTCGGG	SybrGreen
NEO	TAACTAGTATGATTGAACAAGATGGATTGCA	TATGCGGCCGCTCAGAAGAACTCGTCAAGAA	CGGTGCCCTGAATGAACTGCA
